# Reaction norm for genomic prediction of plant growth: modeling drought stress response in soybean

**DOI:** 10.1007/s00122-024-04565-5

**Published:** 2024-03-09

**Authors:** Yusuke Toda, Goshi Sasaki, Yoshihiro Ohmori, Yuji Yamasaki, Hirokazu Takahashi, Hideki Takanashi, Mai Tsuda, Hiromi Kajiya-Kanegae, Hisashi Tsujimoto, Akito Kaga, Masami Hirai, Mikio Nakazono, Toru Fujiwara, Hiroyoshi Iwata

**Affiliations:** 1 Graduate School of Agricultural and Life Sciences, The University of Tokyo, Tokyo, Japan; 2 Arid Land Research Center, Tottori University, Tottori, Japan; 3 Graduate School of Bioagricultural Sciences, Nagoya University, Nagoya, Japan; 4 Tsukuba-Plant Innovation Research Center (T-PIRC), University of Tsukuba, Tsukuba, Japan; 5 Research Center for Agricultural Information Technology, NARO, Tokyo, Japan; 6 Institute of Crop Science, NARO, Tsukuba, Japan; 7 RIKEN Center for Sustainable Resource Science, Tsukuba, Japan

## Abstract

**Key message:**

We proposed models to predict the effects of genomic and environmental factors on daily soybean growth and applied them to soybean growth data obtained with unmanned aerial vehicles.

**Abstract:**

Advances in high-throughput phenotyping technology have made it possible to obtain time-series plant growth data in field trials, enabling genotype-by-environment interaction (G × E) modeling of plant growth. Although the reaction norm is an effective method for quantitatively evaluating G × E and has been implemented in genomic prediction models, no reaction norm models have been applied to plant growth data. Here, we propose a novel reaction norm model for plant growth using spline and random forest models, in which daily growth is explained by environmental factors one day prior. The proposed model was applied to soybean canopy area and height to evaluate the influence of drought stress levels. Changes in the canopy area and height of 198 cultivars were measured by remote sensing using unmanned aerial vehicles. Multiple drought stress levels were set as treatments, and their time-series soil moisture was measured. The models were evaluated using three cross-validation schemes. Although accuracy of the proposed models did not surpass that of single-trait genomic prediction, the results suggest that our model can capture G × E, especially the latter growth period for the random forest model. Also, significant variations in the G × E of the canopy height during the early growth period were visualized using the spline model. This result indicates the effectiveness of the proposed models on plant growth data and the possibility of revealing G × E in various growth stages in plant breeding by applying statistical or machine learning models to time-series phenotype data.

**Supplementary Information:**

The online version contains supplementary material available at 10.1007/s00122-024-04565-5.

## Introduction

Improved measurement techniques have enabled time-series measurements of crop phenotypes in the laboratory and the field (Furbank and Tester 2011; Cabrera-Bosquet et al. 2012; Araus and Cairns 2014). Because crop production can be considered the accumulation of genetic and environmental effects during its growth process, analyzing factors affecting the crop growth process is essential for genetic improvement and cultivation management. In light of current and future climate change, it is crucial to understand and utilize the pattern of genotypes by environmental effects (G × E) for crops’ genetic improvement and cultivation management (Cooper et al. 2021). However, the mechanism of how and at which stage of crop growth G × E occurs and how it contributes to the final product remain unclear. Data analysis methods that help elucidate this mechanism will significantly contribute to developing varieties that sustain stable crop production in future environments.

Genomic prediction models are now the basis for modeling the relationships among the genome, environment, and growth processes. In recent years, crop breeding has taken advantage of inexpensive high-throughput genotyping via genomic selection (Meuwissen et al. 2001), essential for accelerating crop breeding (Heffner et al. 2009). Various genomic prediction models incorporating G × E predictions have also been proposed for adaptation to future environments through genomic selection (Burgueño et al. 2012; Schulz-Streeck et al. 2013; Jarquin et al. 2014; Technow et al. 2015; Pierre et al. 2016).

The model proposed by Jarquin et al. (2014) reflects the idea of the reaction norm, which is the pattern of phenotypic change exhibited by an individual under different environmental conditions. For example, if the reaction norm for drought stress is known for the yield of a given variety, it is possible to predict whether the variety will maintain a stable yield under future drought stress conditions. The proposed model can incorporate the reaction norm as a random effect and has been applied in various studies (Pierre et al. 2016; Junior et al. 2018; Adhikari et al. 2020; Persa et al. 2020; Jarquin et al. 2021). If the estimation of the reaction norm can be applied to time-series data, it will provide important clues for determining the stage at which G × E transitions occur in the crop.

Random regression, employed in animal quantitative genetics, is an effective method for estimating reaction norms in time-series data. Random regression is a mixed model that includes covariates transformed with basis functions as explanatory variables, which enables the estimation of continuous changes in the effects of covariates such as time. The model proposed by Brugemann et al. (2011) can treat additive genetic effects and permanent environmental effects as functions of both time and heat stress and has been applied to the milk yield of dairy cows (Bohlouli et al. 2013; Santana et al. 2016). These studies have successfully disentangled the genetic, environmental, and G × E effects in time-series data and are expected to be applied to crop growth data. However, there are two issues with using this model for crop growth because of the differences in the characteristics of milk yield data and crop growth data.

The first is the characterization of the effects of environmental stress on the growth process. In the milk yield model, the effects of time and heat stress were assumed to be additive, and their interactions were not considered. In other words, it is assumed that if the milk yield is reduced by temporal heat, it will quickly recover when the heat subsequently subsides. However, the effects of environmental stresses on crop growth may persist. For example, individuals whose growth is delayed by a temporary water shortage remain smaller than those without stress. The second issue, which may be more severe than the first, is the existence of different growth stages. In the case of milk yield, it was assumed that the effect of environmental stress does not change in a time-dependent manner because they deal with individuals that have grown large enough to be milkable. However, during the growth process of a crop, both the size and architecture of plants evolve significantly depending on the growth stage, leading to temporal changes in environmental effects. For example, the effect of drought stress may change with increasing water absorption capacity as the roots grow.

Considering these issues, we propose a novel model that considers reaction norms in crop growth. One of the main features of this model is that daily growth is the response variable. This feature allows the model to consider the characteristics of environmental effects on crop growth. The model also included the number of days after sowing (DAS) as an explanatory variable to account for temporal changes in the reaction norms. We applied the proposed model to analyze soybean growth data obtained by remote sensing using an unmanned aerial vehicle (UAV-RS) to verify its performance. Several drought treatments were implemented in the soybean field trial, and soil moisture content was measured during growth. The data obtained from the trial were used to estimate the response of soybean growth to soil moisture content, demonstrating the potential of the proposed method for evaluating the predictive performance of plant growth of untested genotypes or in response to untested environments.

## Materials and methods

### Plant materials and field trials

We used 198 soybean accessions, most of which (192 accessions) were from Japanese and world soybean mini-core collections of NARO Genebank (https://www.gene.affrc.go.jp/index_en.php). In addition, an Indian cultivar ‘L323’ (JP241838) and a Japanese cultivar ‘Misuzudaizu’ (JP28856) were obtained from NARO Genebank, Japanese landrace ‘Houjaku Kuwazu’ (PI416937) and a United States (US) cultivar ‘5002 T’ (PI634193) were obtained from the USDA (United States Department of Agriculture) germplasm collection through GRIN (Germplasm Resources Information Network), and a soybean cultivar ‘Norin2’ and a Glycine soja accession (B01167) were obtained from the National BioResource Project (https://www.legumebase.brc.miyazaki-u.ac.jp).

**Fig. 1:**
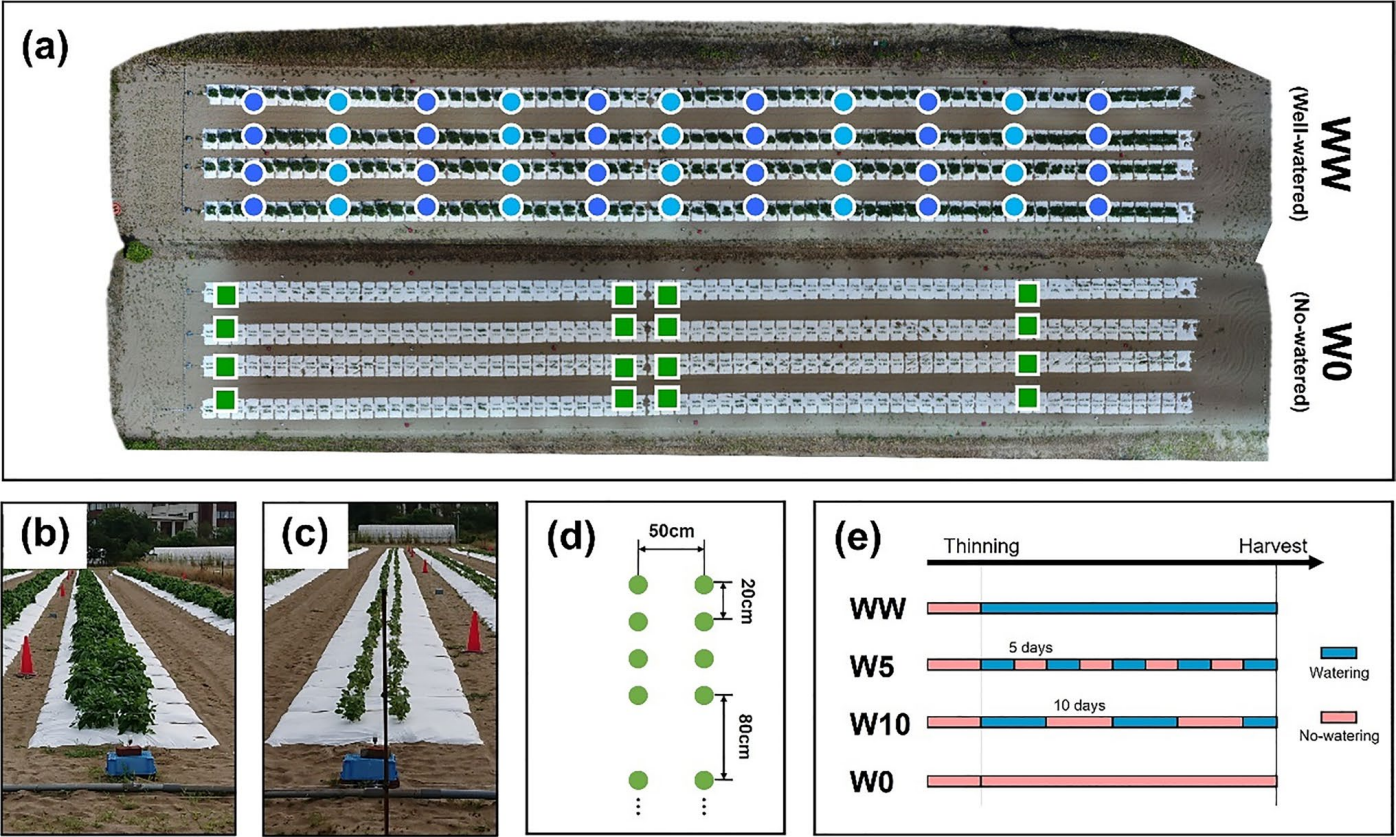
Explanation of the field experiments. **a** An ortho-mosaic image of the field obtained on August 25, 2018. The ortho-mosaic images were created for each treatment (WW/W0). Blue circles indicate measurement points of soil moisture. Colors represented groups of points that were measured alternately. Green squares indicate plots in which the height of plants was measured manually. Circles and squares are drawn in fields of WW and W0, respectively, but soil moisture and plant height were measured in the same pattern of plots. **b, c** Ground-level images of treatments WW and W0. **d** Planting pattern of plots made of two rows of four plants (green dots) and separated by 80 cm. **e** Schematic illustration of watering patterns of four treatments

Field trials and data acquisition were the same as in a previous study (Toda et al. 2022), except for the watering treatments. From 2017 to 2019, the field trial was conducted in an experimental field with sandy soil at Arid Land Research Center, Tottori University (35°32′ N lat, 134°12′ E long, 14 m above sea level). In 2018‒2019, 198 accessions were used, whereas 186 of 198 accessions were used in 2017. Each plot consisted of four plants, and the distances between rows, plots, and plants were 50, 80, and 20 cm, respectively (Fig. 1d). In all years, sowing was performed at the beginning of July, followed by thinning after two weeks. Fertilizers (15 g m^−2^, 6.0 g m^−2^, 20 g m^−2^, 11 g m^−2^, and 7.0 g m^−2^ of N, P, K, Mg, and Ca, respectively) were applied to the field before sowing.

In 2017 and 2018, two watering treatment levels, well-watered (WW) and non-watered (W0), were used to evaluate the genetic variations in response to different environmental conditions. In 2019, four watering levels were investigated including WW and W0. Two other treatments, five days of watering followed by five days of no watering (W5) and ten days of watering followed by ten days of no watering (W10), were also included. White mulch sheets (Tyvek, DuPont, US) were laid to prevent rainwater infiltration and to control soil conditions with artificial irrigation. Watering tubes were installed under the sheets to irrigate the fields. Artificial irrigation was applied for five hours daily (7:00‒9:00, 12:00‒14:00, and 16:00‒17:00) starting the day after thinning in the watering treatments. The following text uses an abbreviation for denoting a specific combination of the treatment level and the experiment’s year; treatment WW in 2017 is abbreviated as ‘2017-WW.’

### Data acquisition

UAV-RS was started after thinning and performed 16–35 times during cultivation. Consumer UAVs (DJI Phantom 4 Advanced, Shenzhen, China) were used for the RGB image collection. A UAV flowed 12–14 m above the ground and captured images at intervals of two seconds with an autofocus function. A single flight of the UAV took approximately 15 min, and 500–600 images were captured for each treatment.

The plant heights of a subset of plots were measured manually as ground truth data and used to correct the UAV-RS-based canopy height measurement bias. Plant height was defined as the distance from the top of the plant to the ground. In 2017, all the plots were separated into three groups, and the plant height of each group was measured almost once a week. In 2018 and 2019, nine and eight plots were chosen as references for each block, respectively (Fig. 1a), and the plant heights of the selected plots were measured every day.

Soil moisture was measured daily using a handheld instrument (TDR-341F; Fujiwara Seisakusho, Japan). We selected 48, 64, and 48 plots as measurement targets for 2017, 2018, and 2019, respectively. The selected plots were divided into several groups (three groups in 2017 and two groups in 2018 and 2019), and measurements were taken each day alternately (Fig. 1a).

### Estimation of the daily canopy area and height

Ortho-mosaic images of the field of each UAV-RS were constructed from the images collected in the UAV-RS using Pix4Dmapper (Pix4D, Switzerland). Next, images of individual plots were segmented from ortho-mosaic images based on the geolocation information identified using ArcGIS (ESRI, US). Each plot’s canopy area was estimated as the area of the canopy projected onto the ortho-mosaic images. The canopy height of each plot was estimated as the difference between the height at the top of the canopy and the average ground altitude. The NDVI-based thresholds were applied to segment the canopy and ground regions in the image of an individual plot. The image analysis process was implemented using Python 3.72, library OpenCV (ver.4.1.0), and GDAL (ver.3.2.2). For the 2019 data, a similar procedure was conducted by Hiphen Inc. (https://www.hiphen-plant.com). The analytical protocol was the same as in previous studies (Verger et al. 2014; Madec et al. 2017).

Because the canopy height data measured with UAV-RS had biases depending on the measurement date, a correction was applied using the height of the plants selected for manual measurement. A simple equation (Toda et al. 2021) was used, in which the UAV-RS canopy height on day *d* and plot *i* (CH_*d,i*_) consisted of the plant height measured manually (PH_*d,i*_), daily bias (*b*_*d*_), and measurement noise (*e*_*d,i*_). After estimating the bias, the corrected values of the canopy height (CH_*d,i*_/*b*_*d*_) were used in the following analysis.

1 $$\begin{array}{*{20}c} {{\text{CH}}_{{d,i}} = b_d {\text{PH}}_{d,i} + e_{d,i} } \\ \end{array}$$

Daily changes in the canopy area and height were estimated using smoothing splines to trace the daily growth process. The following analyses were performed using R software (ver. 4.1.3) (R Core Team 2022). Parameter ‘lambda’ of function ‘smoothing.spline’ was set as 0.0001 and 0.001 for canopy area and height, respectively.

### Interpolation of soil moisture

Because soil moisture was measured in limited locations and on limited dates, soil moisture in all plots and dates was estimated using model-based interpolation. The spatial and temporal similarities in soil moisture were modeled using the kernel method.

2 $$\begin{array}{*{20}c} {y_{i,j,d} = \frac{1}{C_{i,j,d}}\mathop \sum \limits_{{j^{\prime } }} \mathop \sum \limits_{{d^{\prime } }} k_{s} \left( {l_{j} - l_{{j^{\prime } }} } \right) k_{t} \left( {d - d^{\prime } } \right)I\left( {i - i^{\prime } } \right) y_{{i^{\prime } ,j^{\prime } , d^{\prime } }} ,} \\ \end{array}$$

where *y*_*i,j,d*_ is the soil moisture data of row *i* and plot *j* on days *d*, *C*_*i*__,__*j*__,__*d*_ is a constant to guarantee that *y*_*i,j,d*_ is a weighted mean of *y*_*i’,j’,d’*_, *k*_*s*_(·) is a kernel function for the spatial effect, *k*_*t*_(·) is the kernel function for the temporal effect, *l*_*i*_ is the location (integer) of plot *i*, and *I*(·) is a function for selecting the same row, where* I*(0) = 1; otherwise, 0. This means that only the soil moisture data measured in the same row were used for interpolation because the soil moisture condition was assumed to be dependent on each watering tube (i.e., independent among different watering tubes). A Gaussian kernel function was used for *k*_*s*_(·) and *k*_*t*_(·), thus

3 $$\begin{array}{*{20}c} {k_{s} \left( x \right) = \exp \left( { - \frac{{x^{2} }}{{\lambda_{s} }}} \right),} \\ \end{array}$$

4 $$\begin{array}{*{20}c} {k_{t} \left( x \right) = \exp \left( { - \frac{{x^{2} }}{{\lambda_{t} }}} \right).} \\ \end{array}$$

The bandwidths of the kernels, *λ*_*s*_ and *λ*_*t*_, were chosen from the candidate values (17 values from 0.1 to 1000) each year and field with leave-one-out cross-validation. Root mean squared errors (RMSE) were used to evaluate the interpolation accuracy in the cross-validation.

### Genomic relationship matrix

Whole-genome sequencing data of all 198 accessions (Kajiya-Kanegae et al. 2021) were used as previously described (Toda et al. 2022). Biallelic SNPs with a minor allele frequency (MAF) ≥ 0.025, missing rate < 0.05, and linkage disequilibrium < 0.95 were employed. Missing genotypes were imputed with Beagle 5.0 (Browing et al. 2018), using default parameter settings. The genome-wide SNP genotype data used in the analysis included 425,858 SNPs. Genotypes of individual alleles were scored as −1 (homozygous for the reference allele), 1 (homozygous for the alternative allele), or 0 (heterozygous for the reference and alternative alleles).

Using the genome data matrix **X** (an *n* × *m* scaled SNP genotype score matrix, where *n* and *m* are the numbers of genotypes and markers, respectively), we estimated the genomic relationship matrix **G** using a linear kernel function (**G**_**L**_):

5 $$\begin{array}{*{20}c} {{\text{G}}_{{{\text{L}}ij}} = \frac{{{\mathbf{x}}_{i}^{{\text{T}}} {\mathbf{x}}_{j} }}{{c_{ij} }}} \\ \end{array}$$

and Gaussian kernel function (**G**_**G**_),

6 $$\begin{array}{*{20}c} {{\text{G}}_{{{\text{G}}ij}} = \exp \left( { - \frac{{\left\| {{\mathbf{x}}_{i} - {\mathbf{x}}_{j} } \right\|^{2} }}{{\lambda_{g} }} } \right)} \\ \end{array}$$

where G_L*ij*_ and G_G*ij*_ are the elements of **G**_**L**_ and **G**_**G**_, respectively, in row *i* and column *j*, **x**_*i*_ is *i*th row vector of **X**, and *c*_*ij*_ is a normalizing constant. These two kernel functions were selected because different types of genetic effects can be considered. Only additive genetic effects on traits are included using **G**_**L**_ as a variance–covariance matrix of random genetic effects (Morota and Gianola 2014), whereas an infinite order of epistasis can be considered using **G**_**G**_ (Jiang and Reif 2015). *λ*_*g*_ = 10^5^ is a kernel bandwidth hyperparameter selected from candidates between 10^5^ and 10^7^. Optimal *λ*_*g*_ was selected based on genomic prediction accuracy of the canopy area of the last observation date, which was evaluated by repeating tenfold cross-validation ten times. An R package, ‘rrBLUP’ (ver. 4.6.1) (Endelman 2011), was used to estimate **G**_**L**_ (Eq. 5).

### Daily growth model

We propose two models that include the effects of genotype, soil moisture, and growth stage, and their interactions on the observed growth curves. One model is the spline (SP) model that expresses daily growth using a statistical model. This model assumes that daily growth is determined by the reaction norm of the soil moisture, which can be represented by a spline curve:

7 $$\begin{array}{*{20}c} {\Delta y_{i, d} = y_{i, d} - y_{i,d - 1} = {\text{SP}}_{ i,d} \left( {s_{i, d - 1} } \right) + e_{i,d} ,} \\ \end{array}$$

where *y*_*i,d*_ is the canopy area or height of plot *i* on day *d*, Δy_*i,d*_ is the difference in canopy area or height between days *d* and *d* ‒ 1, and SP_*i,d*_(·) is the spline function for plot *i* on day *d*, *s*_*i,d‒*1_ is the soil moisture of plot *i* on day *d* ‒1, and *e*_*i,d*_ is the residual term. We prepared different reaction norm curves (SP_*i,d*_) for each plot (*i*) and growth stage (*d*). Because a linear connection of the basis functions can describe the spline function,

8 $$\begin{array}{*{20}c} {{\text{SP}}_{i,d} \left( {s_{d - 1} } \right) = \mathop \sum \limits_{q = 1}^{Q} c_{q,i,d} \phi_{q} \left( {s_{i, d - 1} } \right),} \\ \end{array}$$

where *Q* is the number of basis functions of the spline, *ϕ*_*q*_(·) is the *q*^th^ basis function, and *c*_*q,i,d*_ is its coefficient. Therefore, we assumed that the coefficients *c*_*q,i,d*_ represented a variety of reaction norms among the genotypes and growth stages. The B-spline basis function is used as *ϕ*_*q*_, and three values (3, 4, and 5) were assigned to the number of basis functions *Q*.

We then modeled the effects of genotype and growth stage on the coefficients *c*_*q,i,d*_. It was assumed that the coefficients of genetically close genotypes were similar, and those on close observation dates were similar. In other words, the coefficients were assumed to vary smoothly among genotypes and dates. Mixed models are one of the standard methods under such an assumption, but for ease of implementation, we focused on a varying coefficient model (Hastie et al. 2009). In this model, minimization of the weighted least squares method was used for the estimation. To consider effects of both genotypes and growth stages, we prepared the following weighted least squares of residuals:

9 $$\begin{array}{*{20}c} {\mathop \sum \limits_{{i^{\prime} = 1}}^{N} \mathop \sum \limits_{{d^{\prime} = 1}}^{D} G_{{i,i^{\prime}}} k_{0} \left( {d - d^{\prime}} \right)e_{{i^{\prime},d^{\prime}}}^{2} ,} \\ \end{array}$$

10 $$\begin{array}{*{20}c} {k_{0} \left( x \right) = \exp \left( { - \frac{{x^{2} }}{{\lambda_{0} }}} \right),} \\ \end{array}$$

where *G*_*i,i’*_ is an element of matrix **G** corresponding to the genotypes of plot *i* and *i’*, *k*_0_(·) is the Gaussian kernel of the observation date, *e*_*i,d*_ is the residual term of Eq. 7 in plot *i* on day *d*, and *λ*_0_ is the kernel width. The estimations of *c*_*q,i,d*_ using the minimization of Eq. 9 were conducted for each combination of plot *i* and date *d*. Data for plot *i’* or day *d’* were weighted based on the genotypic similarity *G*_*i,i’*_ and closeness of dates *k*_0_(*d ‒ d’*) when estimating the daily growth of plot *i* or day *d*; thus, data from similar genotypes and growth stages were automatically chosen for the estimation. **G**_**L**_ and** G**_**G**_ were assigned to **G**, but for ease of computation, zeros were assigned to elements of **G**_**L**_ if they were less than zero. Four values (10, 30, 50, and 70) were assigned to the kernel bandwidth *λ*_0_. In context of random regression, this model corresponds to an assumption cov(**c**_*q*_) = *σ*_*q*_^2^(**Z**_K_**KZ**_K_^t^) ○ (**Z**_G_**GZ**_G_^t^), where **c**_*q*_ is a vector of length *ND* containing *c*_*q,i,d*_ (*i* = 1, …, *N*; *d* = 1, …, *D*), *σ*_*q*_^2^ is a variance of **c**_*q*_, **K** is a *D* × *D* variance–covariance matrix in which elements are *k*_0_(*d* – *d’*), ○ represents Hadamard product, and **Z**_K_ and **Z**_G_ are design matrices.

Another model is the random forest (RF) model (Breiman 2001), where daily growth is modeled with machine learning using genotype, soil moisture, and growth stages as predictors.

11 $$\begin{array}{*{20}c} {y_{i, d} = \text{RF}\left( {y_{i,d - 1} ,{\mathbf{g}}_{i} ,s_{i,d - 1} , d} \right) + e_{i,d} ,} \\ \end{array}$$

where RF(·) is a function of RF, **g**_*i*_ is a column vector of the genomic relationship matrix **G** corresponding to the genotype in plots *i*, and *s* is a measure of the soil moisture. Thus, this model included the effects of genotype (**g**_*i*_), environment (*s*_*i*,*d*‒1_), growth stage (*d*), and their interactions. The column vector of **G** was used to represent the genotypic effect instead of whole-marker genotype data because the original data were too large to be evaluated. Since each column of **G** indicates the genetic relationship between one genotype and the other genotypes, we assumed that column vectors of **G** could be used as an explanatory variable representing position of each genotype in the target population. As for the spline model, **G**_**L**_ and **G**_**G**_ were assigned to **G**. In addition, unlike the SP model of which response variable was daily growth, the response variable is a phenotype itself (*y*_*i,d*_), not its difference, and phenotype one day before (*y*_*i,d*‒1_) was included as a predictor. This structure was chosen to account for the possibility that daily growth is determined by the size of the plant itself by taking advantage of the flexibility of machine learning. An R Package ‘ranger’ (ver. 0.13.1) (Wright and Ziegler 2017) was used to create a random forest model with six values (5, 10, 15, 20, 25, 30) assigned to parameter ‘mtry.’

### Cross-validation and benchmark models

**Fig. 2:**
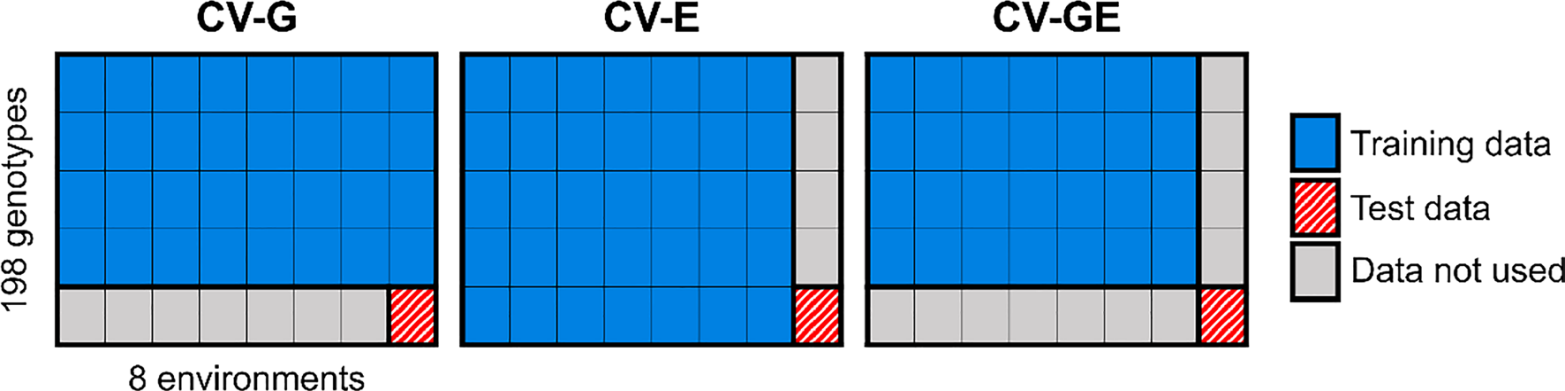
Illustration of patterns of cross-validations. Data usage when predicting growth of trait of one genotype group in one environment is shown. An environment corresponds to one combination of treatment and year (e.g., 2017-WW)

We assessed model performance using the prediction accuracy for three cross-validation patterns (Fig. 2): cross-validation among genotypes (CV-G), cross-validation among environments (CV-E), and cross-validation among both genotypes and environments (CV-GE). In CV-G, fivefold cross-validation applied to 198 genotypes was repeated ten times, and the mean prediction accuracy was used for evaluation. In CV-E, leave-one-environment cross-validation was applied, in which a combination of treatment and year (e.g., 2017-WW) was eliminated from the dataset as an environment, and the remaining combinations (environments) were used for model training. The eliminated combinations (environments) were used to validate the prediction accuracy of the trained model. CV-GE is a combination of CV-G and CV-E, in which all data were split into 40 groups (5 genotype groups × 8 environments). One genotype group and one environment were eliminated from the dataset, and the remaining 28 groups (4 genotype groups × 7 environments) were used for model training. Data from the combination of the eliminated genotype group and the environment were then used to validate the prediction accuracy of the trained model. Thus, in CV-GE, no data on the test genotype or environment are available for training. As in CV-G, CV-GE was repeated ten times with different grouping patterns of genotypes, and mean prediction accuracy was used for evaluation. The model accuracy was evaluated using the correlation coefficients between the predicted and interpolated values.

In these cross-validations, we did not use any observed phenotypic data from the validation dataset. Therefore, we predicted growth for the entire observation period by sequencing the calculations, in which the predicted value for one day was used to predict the phenotype for the next day. The predicted phenotype on day *d* ‒ 1 (${\widehat{y}}_{i, d-1}$) is assigned to the observed phenotype on day *d* ‒ 1 (*y*_*i*,*d*‒1_) using Eqs. 7 and 11 to calculate phenotype of day *d* (${\widehat{y}}_{i, d}$):

12 $$\begin{array}{*{20}c} {\hat{y}_{i, d} = \hat{y}_{i,d - 1} + {\text{SP}}_{ i,d} \left( {s_{i, d - 1} } \right) ,} \\ \end{array}$$

13 $$\begin{array}{*{20}c} {\hat{y}_{i, d} = \text{RF}\left( {\hat{y}_{i,d - 1} ,\mathbf{g}_{i} ,s_{i,d - 1} , d} \right) .} \\ \end{array}$$

A mixed model was used to predict phenotypes on the first observation day. For the CV-G, a simple genomic prediction model was applied to each environment (combination of year and treatment):

14 $$\begin{array}{*{20}c} {{\mathbf{y}}_{h} = \mu_{h} + {\mathbf{Z}}_{h} {\mathbf{u}}_{h} + {\mathbf{e}}_{h} } \\ \end{array}$$

where **y**_*h*_ is a vector of phenotypic values of the first observation day of environment *h*, *μ*_*h*_ is a mean, **Z**_*h*_ is a design matrix, **u**_*h*_ is a vector of random effect of genotypes which follows to a multivariate normal distribution *N*(**0**, *σ*_g_^2^**G**) where *σ*_g_^2^ is a genetic variance and **G** is the genomic relationship matrix, and **e**_*h*_ is a vector of residuals. In CV-E and CV-GE, a mixed model including all the environments was used because the test environment data were not available for training:

15 $$\begin{array}{*{20}c} {\mathbf{y} = \mathbf{X} \boldsymbol{\beta} + \mathbf{Z}\mathbf{u} + \mathbf{e} } \\ \end{array} ,$$

where **y** is a vector of phenotypic values of the first observation day across all environments, **X** and **Z** are design matrices, ***β*** is a vector of fixed effects of environments, **u** is a vector of random effect of genotypes, and **e** is a vector of residuals. Since the data of test genotypes were available in CV-E, the genomic relationship matrix was not used: **u** ~ *N*(**0**, *σ*_g_^2^**I**), where **I** is a diagonal matrix. On the other hand, genomic prediction was applied in CV-GE by using the genomic relationship matrix; **u** ~ *N*(**0**, *σ*_g_^2^**G**). After assigning the training data to **y**_h_ in Eq. 14 and **y** in Eq. 15, the estimated values of **u**_*h*_ in Eq. 14 and **u** in Eq. 15 are used as the predicted values on the first observation day. An R package ‘lme4’ (ver. 1.1–29) (Bates et al. 2015) is used to solve Eq. 15 in CV-E and ‘BGLR’ (ver. 1.1.0) (Pérez and Campos 2014) is used to solve Eq. 14 in CV-G and Eq. 15 in CV-GE. For ‘BGLR’ function in ‘BGLR’ package, the number of total and burn-in MCMC samples (parameters ‘nIter’ and ‘burnIn’) were set at 3000 and 1000, respectively.

The prediction accuracies of the two single-trait genomic prediction models were calculated for comparison. The structures of the models were the same as those on the first day; Eq. 14 for CV-G and Eq. 15 for CV-E and CV-GE, and these models were applied to phenotypic data on each observation day. One of the two genomic relationship matrices (**G**_**L**_ and **G**_**G**_) was selected based on accuracy and used as a variance–covariance matrix of random effects of genotypes (**u**_*h*_ and **u**) in all CV. The two genomic prediction models differ in their response variables. Genomic prediction (GP) used daily phenotypes as a response variable, whereas genomic prediction of growth (GPG) used daily growth of phenotypes as a response variable. Therefore, while GP is a simpler model, GPG mimics the structure of the proposed models. However, the prediction accuracies of the GP and GPG were almost the same (Fig. S1), thus only the results of the GPG model are shown in the Results section.

## Results

### Interpolation of the canopy area and height

**Fig. 3:**
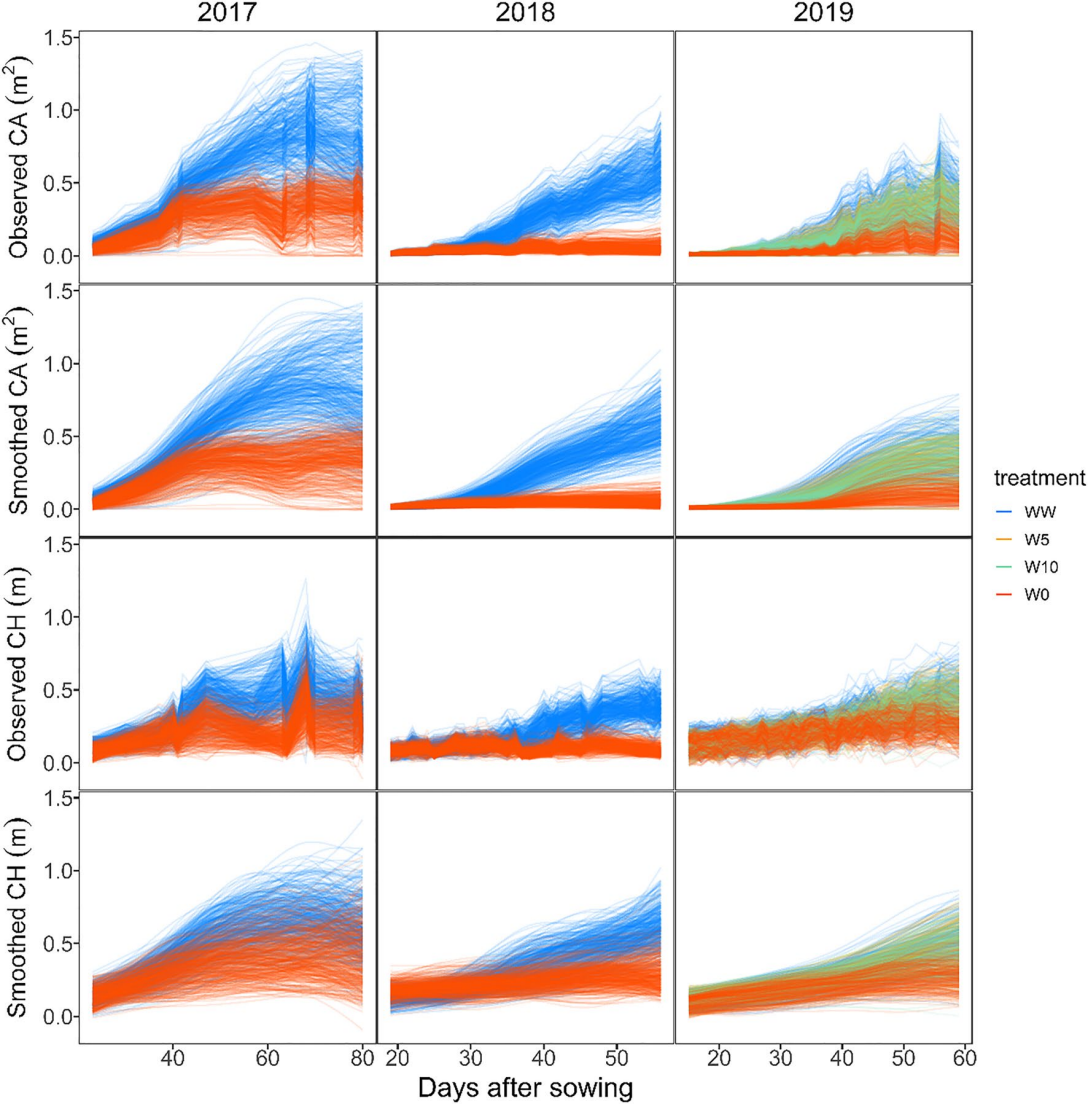
Observed and interpolated values of the canopy area and height. Values of each treatment were plotted in different colors

Estimates of daily changes in the canopy area and height of each plot were calculated by interpolating the UAV-RS measurements (Fig. 3). In all years, the effects of different irrigation patterns on growth were visible. Because temperatures rose considerably in 2018 and caused strong drought stress under the drought treatment, growth in 2018-W0 tended to be lower than in other years. In 2017-W0, a response pattern (two-peaked curve) was estimated in the canopy area, which was unusual. However, no such tendency was found for the canopy height, which was calibrated (Eq. 1) using manually measured values.

### Interpolation of soil moisture data

**Fig. 4:**
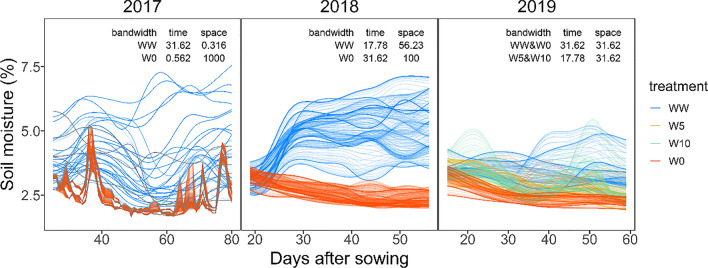
Interpolated values of soil moisture. The soil moisture of each plot was drawn in different lines. Lines of different treatments were drawn in different colors. Bandwidths used for interpolation were written in the figures

The results of soil moisture interpolation based on kernel regression are shown in Fig. 4. The bandwidths for time and space selected using cross-validation were similar in 2018 and 2019. In 2017-WW, the bandwidth for space was small, and the estimation behaved as the nearest-neighborhood estimation for the distribution within the field. Conversely, in 2017-W0, the bandwidth for the time was small, and fine daily variations were reflected in the interpolation results.

### Prediction of daily growth

**Fig. 5:**
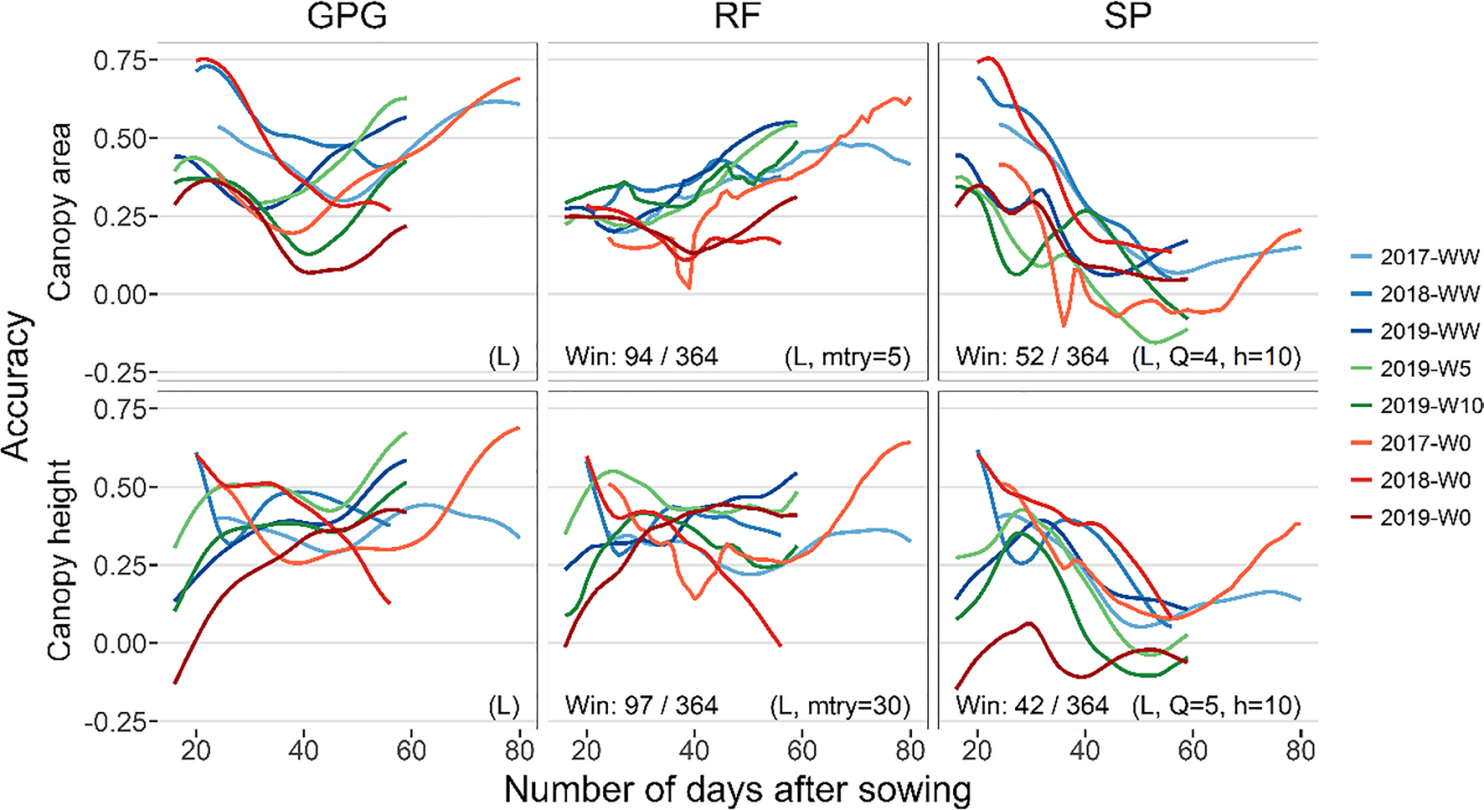
Prediction accuracy of the canopy area and height in cross-validation of genotypes (CV-G). Correlation coefficients of interpolated daily values (Fig. 3) and predicted values were plotted for each day and environment. The top and bottom rows showed the canopy area and height results, respectively. Results of the genomic prediction of growth (GPG), random forest model (RF), and spline model (SP) were arranged along columns. The accuracy of different years and water treatments were plotted in different colors. To compare prediction accuracy, the number of times accuracy of the proposed models exceeded those of GPG was counted and noted in lower left of each panel. Hyperparameters (selection of genomic relationship matrix from **G**_**L**_ or **G**_**G**_ for all models, mtry for RF, *Q* and *h* for SP) with the highest mean prediction accuracy were used, which were shown in bottom right of each panel

**Fig. 6:**
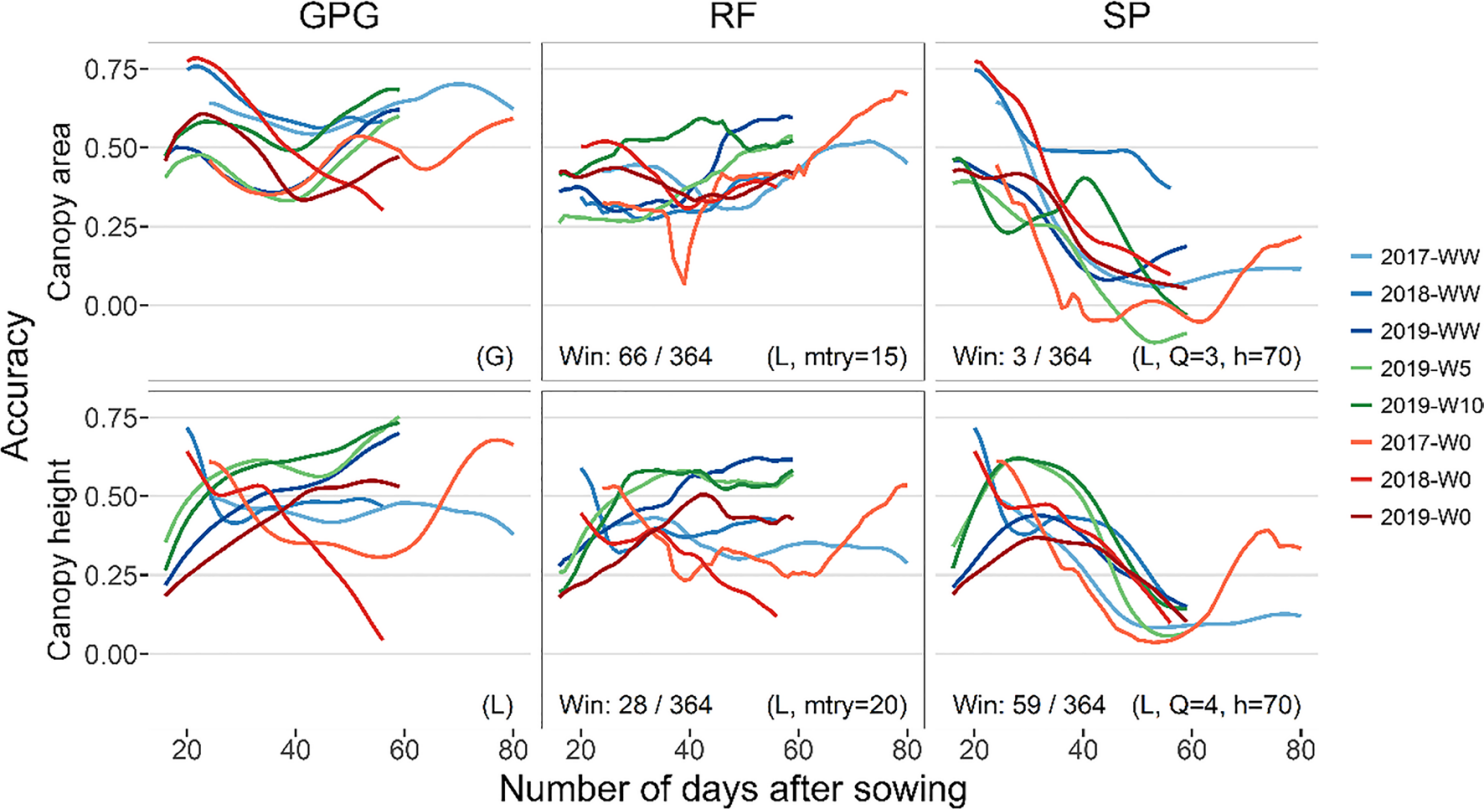
Prediction accuracy of the canopy area and height in cross-validation of environments (CV-E). Details are the same as Fig. 5

**Fig. 7:**
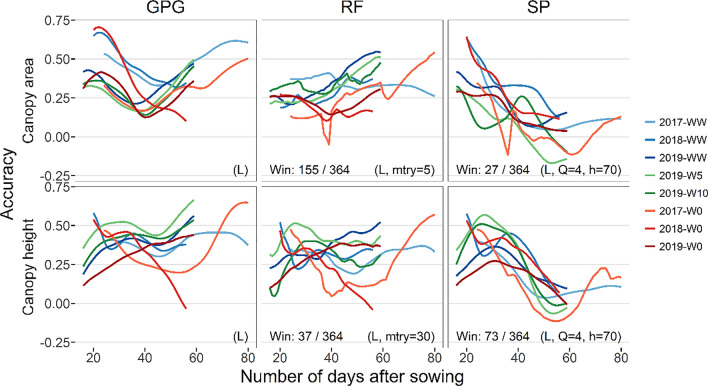
Prediction accuracy of the canopy area and height in cross-validation of both genotypes and environments (CV-GE). Details are the same as Fig. 5

The results were compared between different hyperparameters for the random forest (Fig. S2–7) and spline models (Fig. S8–13). Comparing the prediction accuracy using the hyperparameters and genomic relationship matrices with the highest prediction accuracy (Figs. 5, 6, and 7), the proposed models were able to outperform GPG only a limited number of times. The accuracy of the proposed models exceeded that of GPG most frequently in CV-GE, in which the random forest model outperformed GPG 155 out of 364 times in the prediction of canopy area, whereas the spline model outperformed GPG 73 out of 364 times in the prediction of canopy height.

Comparing within the random forest model, the accuracy showed an increasing tendency along the date for all CV, especially for the canopy area. On the other hand, the accuracy of the spline model decreased as the number of days increased, except for the canopy height, until 30 DAS in 2019.

### Estimation of reaction norm

**Fig. 8:**
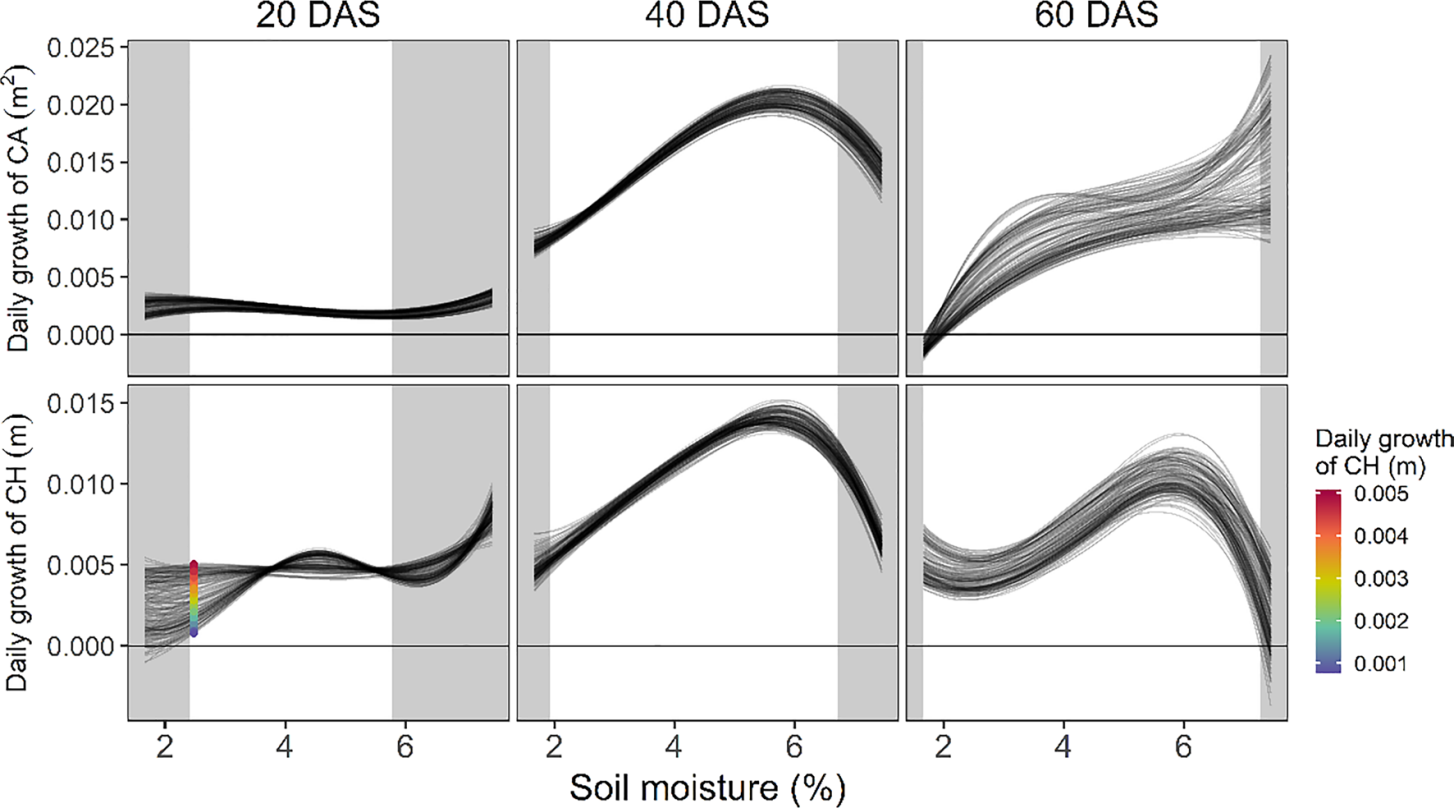
Estimated reaction norm of the canopy area and height. Results of 20, 40, and 60 days after sowing (DAS) are selected. The hyperparameters were set at (*Q*, *h*) = (3, 10) for the canopy area and (4, 10) for the canopy height. Each line corresponds to the reaction norm of daily growth of each genotype. Areas with gray shadows indicate soil moisture ranges observed before and after five days. The canopy height with the greatest variation of the soil moisture (= 2.5% on 20 DAS) is colored. This color is also used in Fig. 9

The estimated reaction norm for daily growth as a function of soil moisture is shown in Fig. 8. The spline model with a linear kernel matrix **G**_**L**_ was chosen because its prediction accuracy was higher than that with **G**_**G**_ (Figs. 5, 6, and 7). While hyperparameter *Q* was set at the same values of those chosen in CV-E (3 for the canopy area and 4 for the canopy height, Fig. 6), *h* was set at 10 for both traits because the estimated reaction norm curves became more varied with time when *h* was set at the smaller value. The estimated environmental response of the daily growth for each genotype was plotted as a curve. Daily growth increased with soil moisture in both canopy area and height. The estimated curves were unstable when the data were not obtained around that day (shadowed area). The estimated daily growth increased from 20 to 40 DAS and decreased from 40 to 60 DAS. On 20 DAS, the estimated genotypic variance in daily growth was more significant for the canopy height than for the canopy area. In particular, there was a large variation in the reaction norms at low soil moisture levels for the canopy height, which will be discussed in detail later.

## Discussion

### Estimation of reaction norms

The proposed method enabled us to estimate the reaction norm of the daily canopy area and height growth concerning soil moisture (Fig. 8). Focusing on the canopy area, the overall growth rate was low at 20 DAS, and there were no significant differences among the genotypes. In contrast, a linear relationship between soil moisture and daily growth rate was observed at 40 DAS. At 60 DAS, the overall growth rate decreased, and differences among genotypes due to differences in flowering phenology appeared. The saturation of growth at high soil moisture levels can be attributed to the increased overlap between leaves, which cannot be measured as an increase in the canopy area. The reaction norm curve was unstable at the periphery of the observed soil moisture content. This is a common phenomenon in spline-function fitting.

**Fig. 9:**
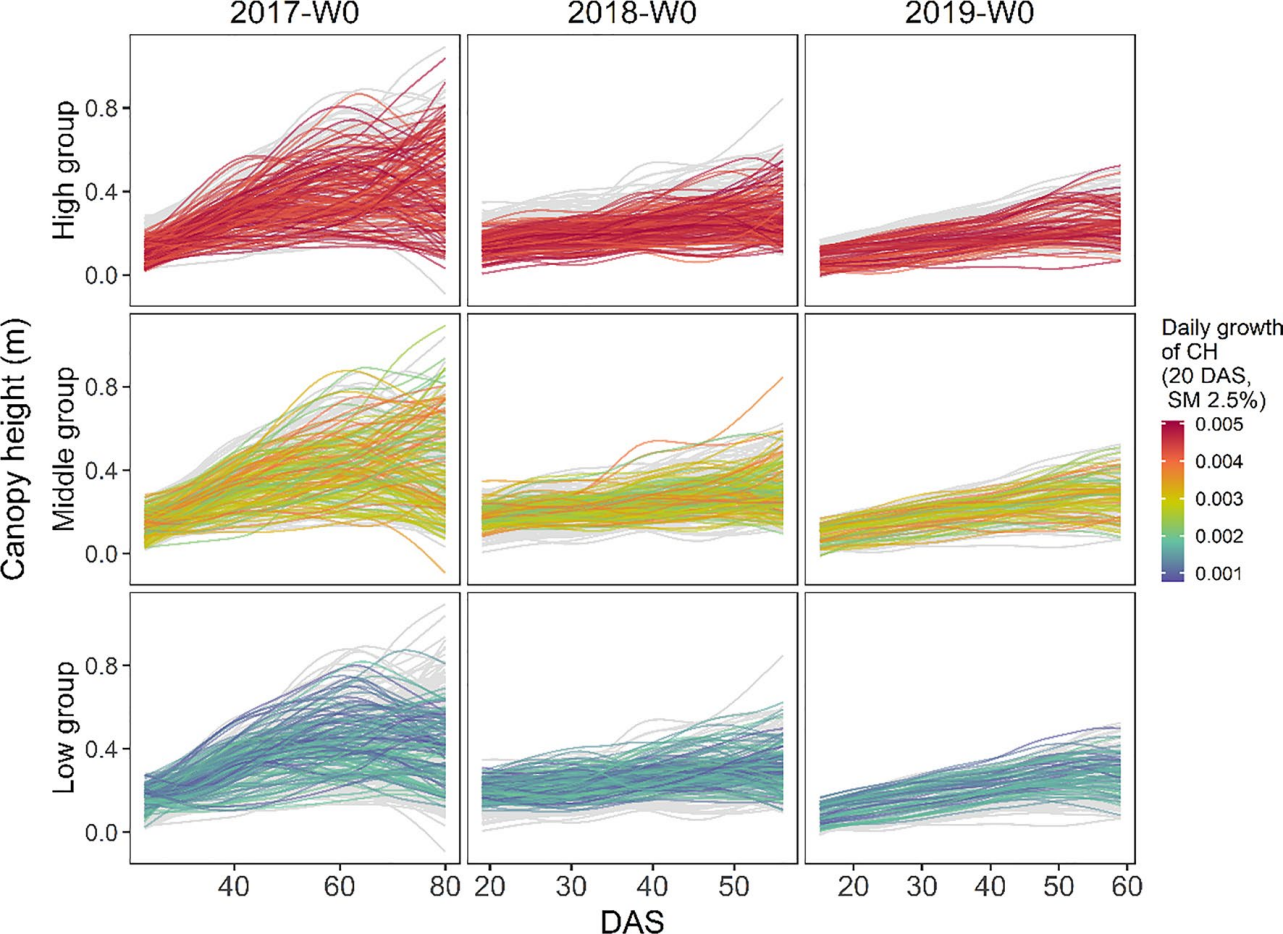
Growth curves of canopy height in W0 treatment, divided according to the coloring in Fig. 8. Genotypes were divided into three groups: high, middle, and low, based on the estimated reaction norms at 2.5% soil moisture on 20 days after sowing (DAS). The curves of all genotypes are drawn in gray, and then the curves for each category are colored to highlight them

Canopy height showed the same trend as the canopy area, with two major differences. Firstly, at 60 DAS, the daily growth dropped with high soil moisture levels. One possible reason is that vigorous growth with rich soil moisture caused lodging. Secondly, at 20 DAS, there was a large genotypic difference in growth when soil moisture was low. Figure 9 shows the actual color-coded growth curves under W0 treatment highlighting this variation in growth. Genotypes with high growth at the point of interest (red) did not have large canopy heights throughout the observation period in 2018 and 2019. Conversely, genotypes with low growth (blue) maintain large canopy height.

One interpretation of this result is that it reflects the growth rate’s effect before the first measurement day. Genotypes that thrived immediately after germination (blue) slowed down their growth speed around 20 DAS because of intense drought stress, but because their plant size at that time was reasonably large, they could receive much solar radiation and became larger individuals during the entire growth period. In contrast, the genotype that slowed its growth speed immediately after germination (red) maintained its growth speed at approximately 20 DAS, reducing the light interception throughout the growth period and leading to small plant size. Following this hypothesis, this method suggests differences in response patterns to drought stress in the early stages of growth. This becomes possible with this method, which models changes in the reaction norm pattern at each growth stage.

### Prediction and model structure

Reaction norm models can be applied to predict the growth curve of a specific environment (i.e., treatment and year combinations) and genotype. From our experiments, we cannot claim that the proposed models surpassed conventional genomic predictions in terms of prediction accuracy. One possible reason for this is that the accuracy of genomic prediction was higher than expected, especially for CV-E and CV-GE. Normally, cross-validation across environments deals with datasets including field trials at different sites, which results in low accuracy of genomic prediction (Jarquin et al. 2017, Vieira et al. 2022). Conversely, because the combinations of year and treatment were treated as environments in this study, simple genomic prediction models could perform well in CV-E and CV-GE by reflecting the high genetic correlation among environments. By validating with datasets in which a larger genotype-by-environment interaction exists, our proposed models may outperform genomic prediction.

Comparing within the proposed models, the prediction accuracy of the random forest model was better than that of the spline model. This suggested a complex interaction between the input variables (plant size, genomic relationships, soil moisture content, and DAS) on the previous day. Machine learning methods specialized for modeling time-series data, such as recurrent neural networks and long short-term memory (Hochreiter and Schmidhuber 1997), may improve prediction accuracy. Additionally, the prediction accuracy of the canopy area in the earlier growth stages tended to be low. This tendency seems to be caused by a shortage of data with small plant sizes; the canopy area at the beginning of the observation period was much smaller than that at later growth stages (Fig. 3). Difficulty in extrapolation is a weakness of machine learning and should be carefully considered in future applications.

For the spline model, the prediction accuracy decreases as the DAS of the prediction target increases. This is because the prediction errors accumulated in the prediction of the time-series changes were ordered from the first to the last day. To overcome this problem, it is necessary to incorporate a structure that considers the entire time-series data simultaneously, such as a state-space model, rather than simply repeating the prediction of the next day. In addition, the prediction accuracy of the canopy area increased from 18 to 30 DAS in 2019. A possible reason for this was the small range of soil moisture during these days (Fig. 4). Because the soil moisture until 30 DAS in 2019 was within a smaller range (2.5–4%) compared with other periods and years, we could supply enough data to estimate the genotype-specific reaction norms of soil moisture in that growth stage. Therefore, if rich growth data under a wider range of soil moisture conditions become available, there may be room to improve the prediction accuracy of the spline model.

To estimate the parameters of the spline model, the criterion of the least weighted sum of squares based on a varying coefficient model was utilized. Compared with the frequently used random regression models, the merits of the varying coefficient model are its ease of implementation and the required computational power. Once the weight is calculated, this model can be implemented using a simple weighted linear regression (e.g., lm function in R). Moreover, although unverified, this simple structure requires less computational power than random regression, which searches for parameters using all data. One major drawback of the varying coefficient model is that the negative elements of the genomic relationship matrix (**G**) must be replaced with zero because the negative weights cannot be treated. Future studies are required to examine the validity of this treatment.

### Application and future improvement

The method proposed in this study enables the estimation of reaction norms using time-series data of plant growth processes. This method is expected to play an important role in the genetic analysis of plant growth subjected to large G × E. Due to climate change, the timing and levels of environmental stress have become diverse. The proposed method, which allows for quantitative evaluation of plant responses to environmental stress during growth, is useful for analyzing important traits such as yield, which is influenced by stress during growth through reduced biomass at harvest.

There are several possible extensions of the method presented in this study. The simplest extension is to use other weather factors as the explanatory variables. However, estimating the reaction norm for each factor is difficult because many weather factors, such as air temperature, usually have only one possible value in a single trial (i.e., no differences among plots). We attempted to estimate the reaction norms of other weather factors, such as air temperature, but the prediction accuracy did not improve. It is necessary to consider them in more straightforward terms, such as fixed effects, to consider weather variables for which the frequency and location of measurements are scarce.

Another possible improvement to our model is the consideration of different growth stages for each genotype. In the present study, we discussed the variation in the growth rate of early canopy height, possibly because the developmental mechanism of the phenotype in the early growth stage is relatively simple. The influence of phenological differences was reflected as growth progressed, making a simple comparison of the reaction norms for the same DAS less meaningful. It is possible to reflect differences in phenology by, for example, replacing the distance of dates (*d* – *d′*) in Eq. 9 with continuous phenological evaluation.

Additionally, data quality and quantity improvements are essential for enhancing the estimation accuracy of the reaction norm. For phenotypic data, UAV-RS and other high-throughput phenotyping methods dramatically increase the quantity of data compared to manual measurements. However, large measurement noise (e.g., Fig. 3) should be suppressed to enhance data quality. In this study, we utilized ground truth data and a smoothing method to estimate actual growth curves; however, other methods, such as real-time kinematic positioning or proximal sensing technologies, could also realize precise measurements. Increasing the quantity of environmental data spatially and temporally is also a topic for future research. While soil moisture measurements were taken manually in this study, introducing automatic measuring devices increased the number of data points and an accurate estimation of the reaction norm significantly.

### Supplementary Information

Below is the link to the electronic supplementary material.

Supplementary file 1 (PDF 2754 KB)

## Data Availability

The datasets generated and/or analyzed during the current study are available from the corresponding author on reasonable request.
