# Supplementary material for: Reaction norm for genomic prediction of plant growth: modeling drought stress response in soybean
Source: Theor Appl Genet. 2024 Mar 9;137(4):77. doi: 10.1007/s00122-024-04565-5 (PMC10924738; doi:10.1007/s00122-024-04565-5)
Supplement: Supplementary file 1 — (PDF 2754 KB) [file 122_2024_4565_MOESM1_ESM.pdf]

Reaction norm for genomic prediction of plant growth: modeling drought stress response in soybean

Yusuke Toda[1], Goshi Sasaki[1], Yoshihiro Ohmori[1], Yuji Yamasaki[1,2], Hirokazu Takahashi[3], Hideki Takanashi[1], Mai Tsuda[4], Hiromi Kajiya-Kanegae[5], Hisashi Tsujimoto[2], Akito Kaga[6], Masami Hirai[7], Mikio Nakazono[3], Toru Fujiwara[1], Hiroyoshi Iwata[1]

[1]Graduate School of Agricultural and Life Sciences, The University of Tokyo, Tokyo, Japan

[2]Arid Land Research Center, Tottori University, Tottori, Japan

[3]Graduate School of Bioagricultural Sciences, Nagoya University, Nagoya, Japan

[4]Tsukuba-Plant Innovation Research Center (T-PIRC), University of Tsukuba, Tsukuba, Japan

[5]Research Center for Agricultural Information Technology, NARO, Tokyo, Japan

[6]Institute of Crop Science, NARO, Tsukuba, Japan

[7]RIKEN Center for Sustainable Resource Science, Tsukuba, Japan

* Correspondence: Hiroyoshi Iwata

hiroiwata@g.ecc.u-tokyo.ac.jp

ORCID: 0000-0002-6949-0795

Supplementary figures

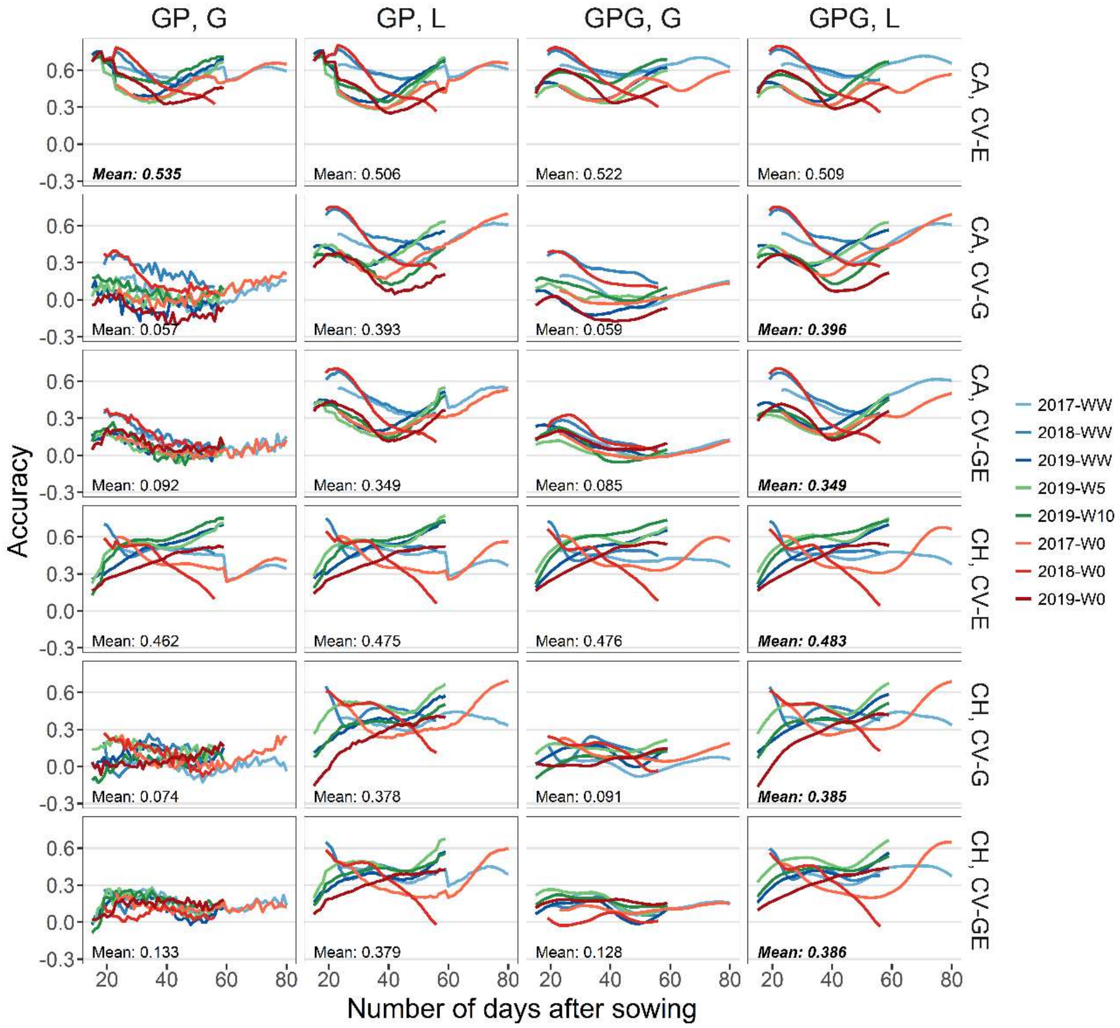

Fig S1 Prediction accuracy of the canopy area (CA) and canopy height (CH) growth using the genomic prediction (GP) and genomic prediction of growth (GPG). Results with different models and genetic relationship matrices (calculated with a linear kernel function: L or Gaussian kernel function: G) are arranged along columns, and results with different traits and cross-validation schemes (CV-G, CV-E, and CV-GE) are arranged along rows. Averages of the accuracy are at the bottom left of each panel. The highest average of accuracy is written in bold and italic font.

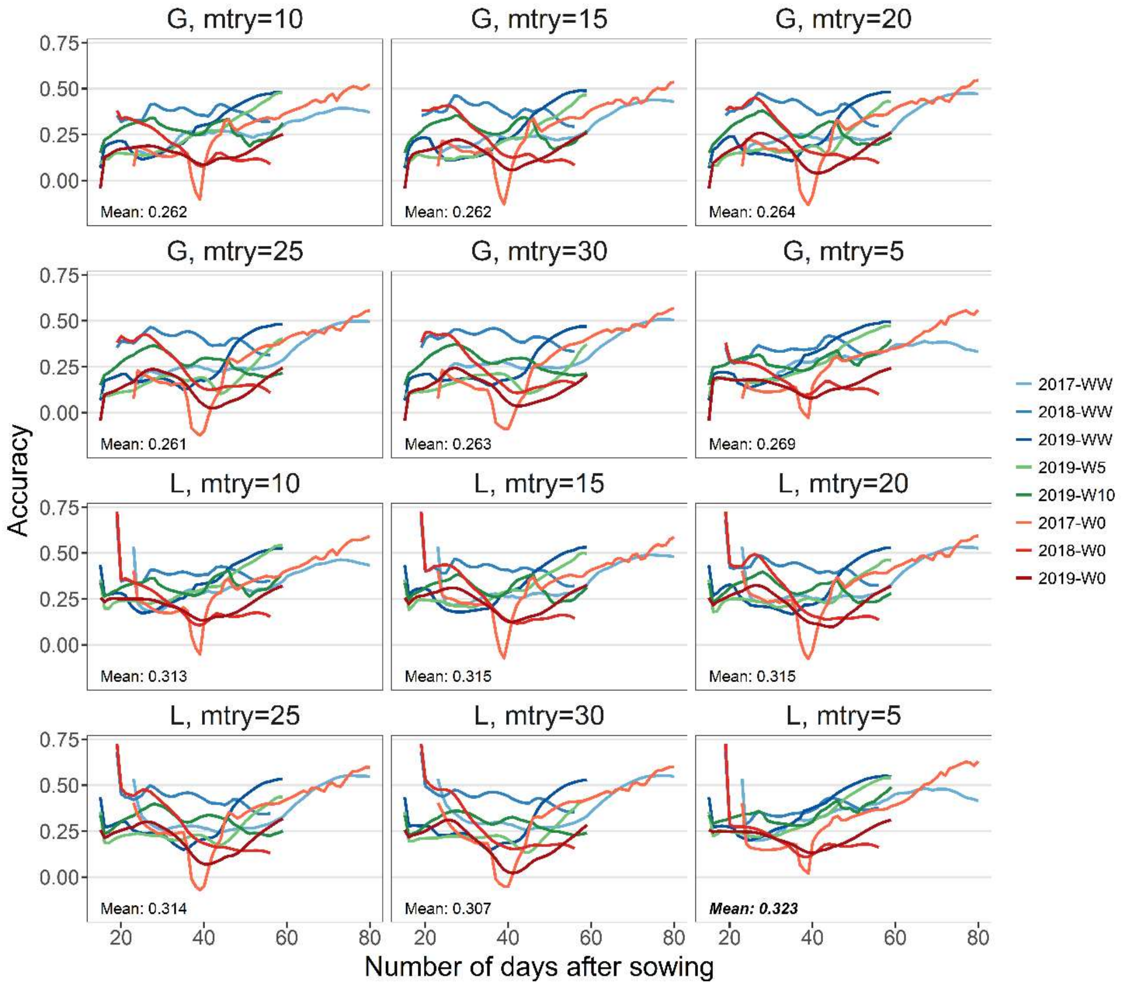

Fig S2 Prediction accuracy of the canopy area growth using the random forest model in cross-validation among genotypes (CV-G). Genetic relationship matrices (calculated with a linear kernel function: L or Gaussian kernel function: G) and a hyperparameter "mtry" used for prediction is written on each panel. Averages of the accuracy are at the top right of each panel. The highest average of accuracy is written in bold and italic font.

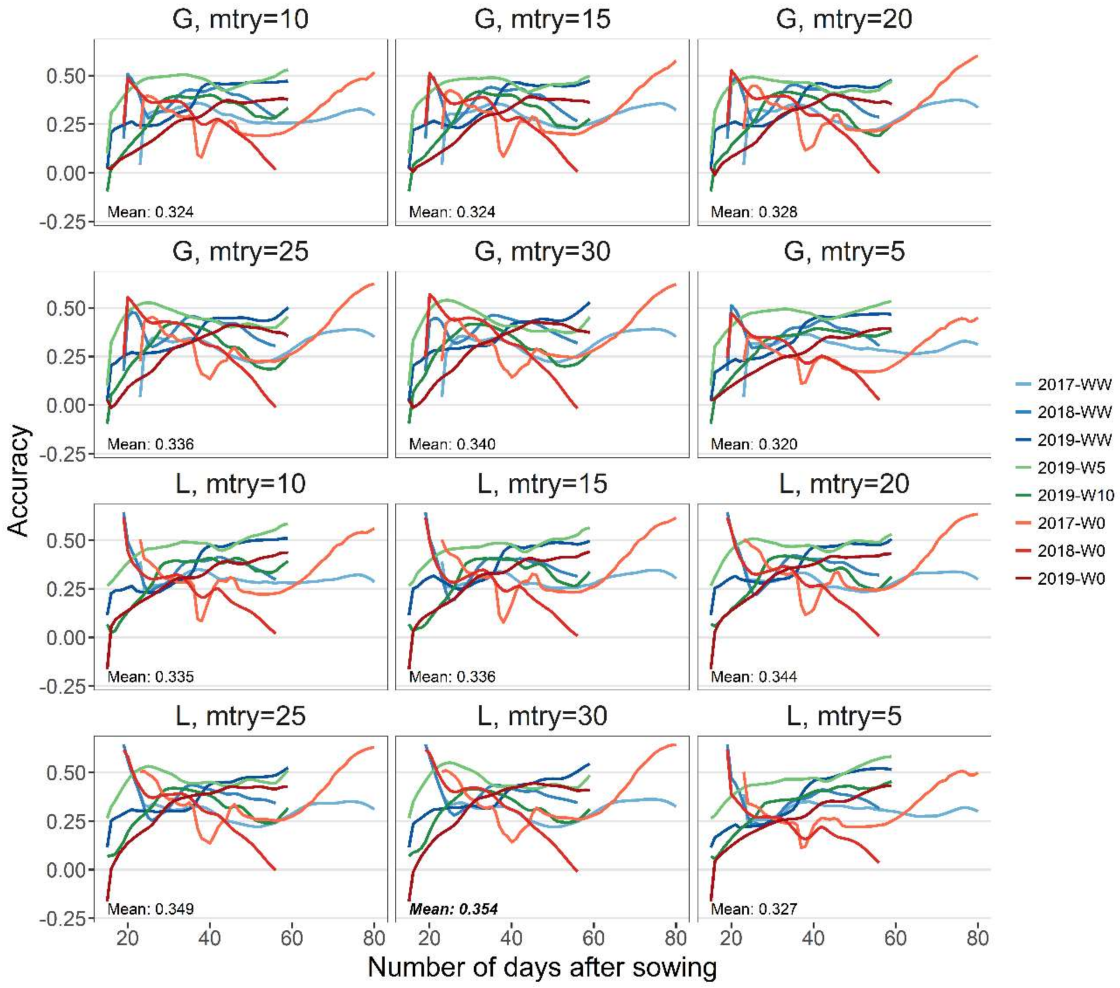

Fig S3 Prediction accuracy of the canopy height growth using the random forest model in cross-validation among genotypes (CV-G). The detail is the same as Fig. S2.

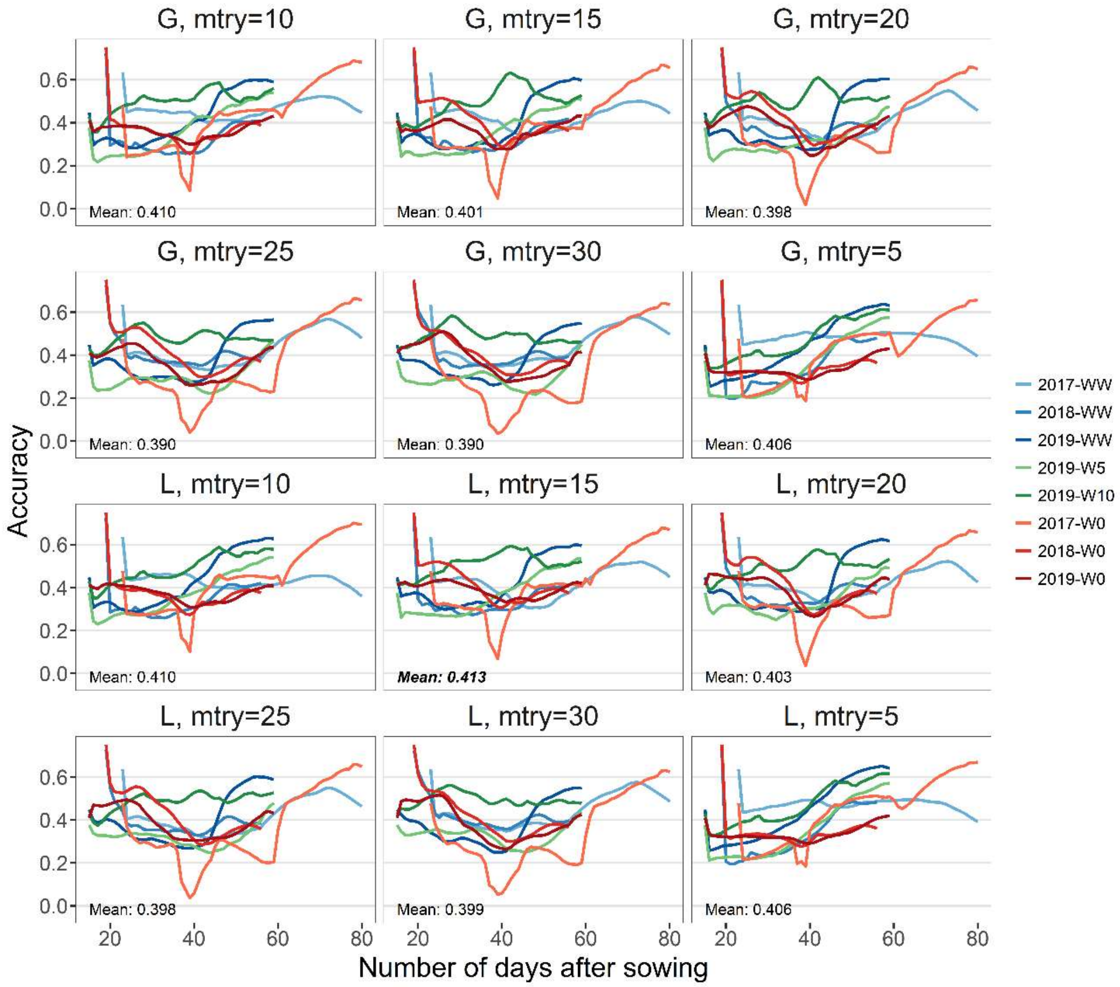

Fig S4 Prediction accuracy of the canopy area growth using the random forest model in cross-validation among environments (CV-E). The detail is the same as Fig. S2.

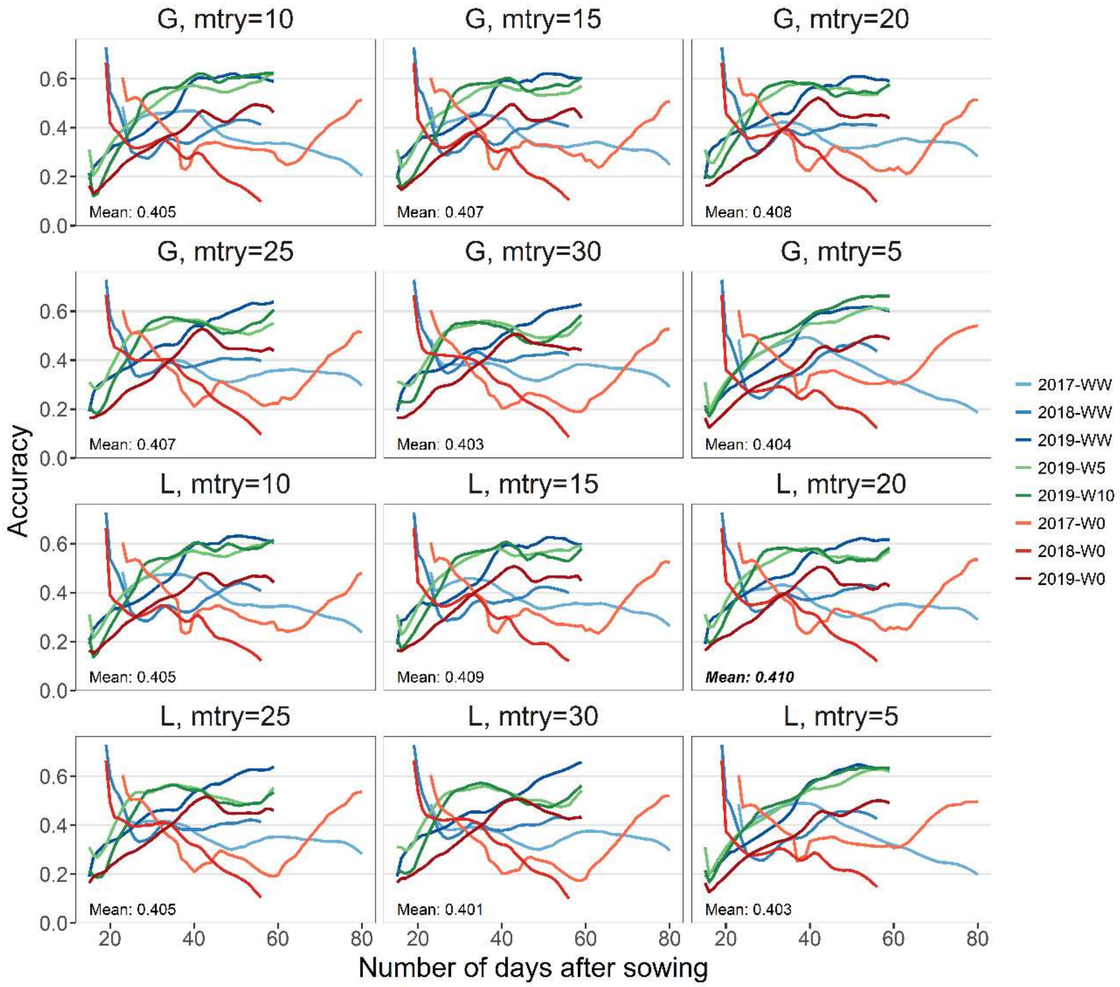

Fig S5 Prediction accuracy of the canopy height growth using the random forest model in cross-validation among environments (CV-E). The detail is the same as Fig. S2.

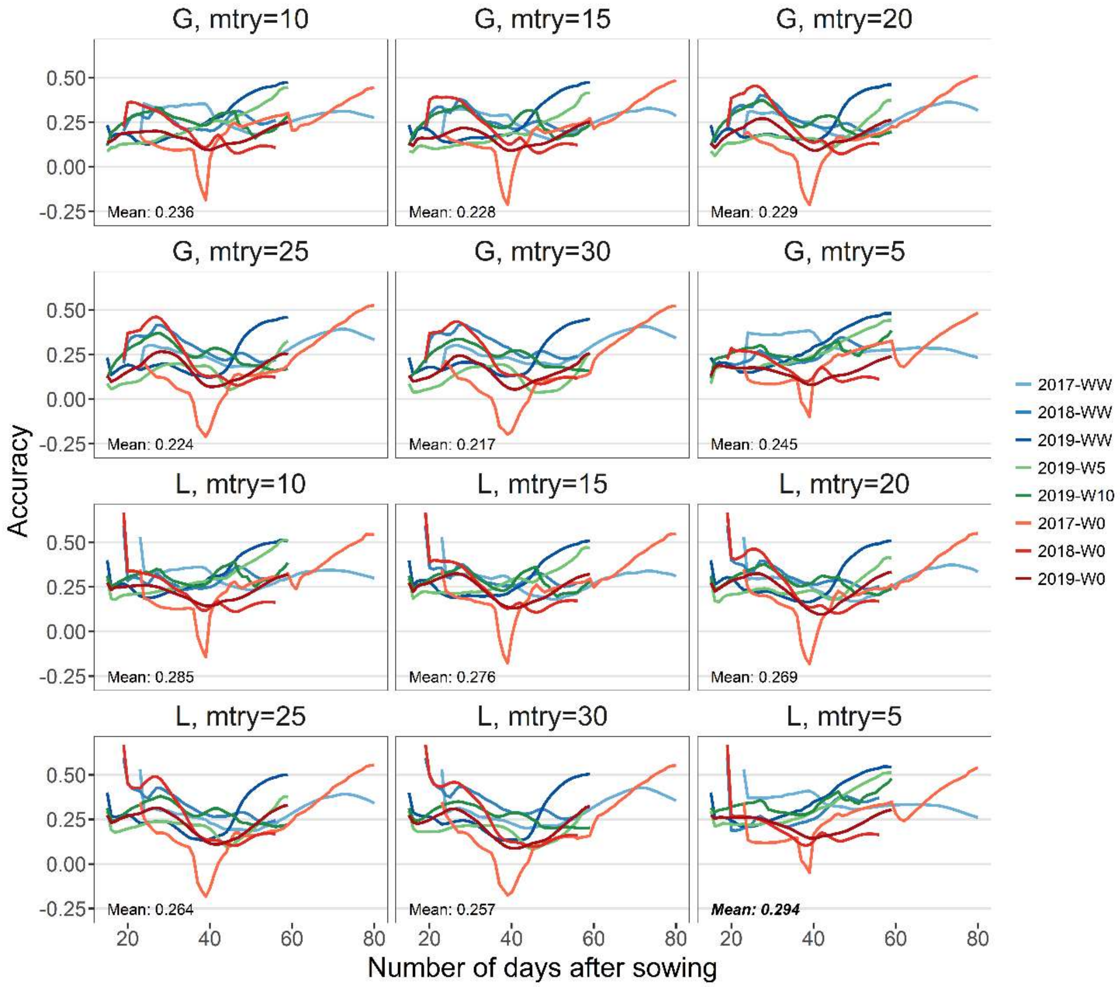

Fig S6 Prediction accuracy of the canopy area growth using the random forest model in cross-validation among genotypes and environments (CV-GE). The detail is the same as Fig. S2.

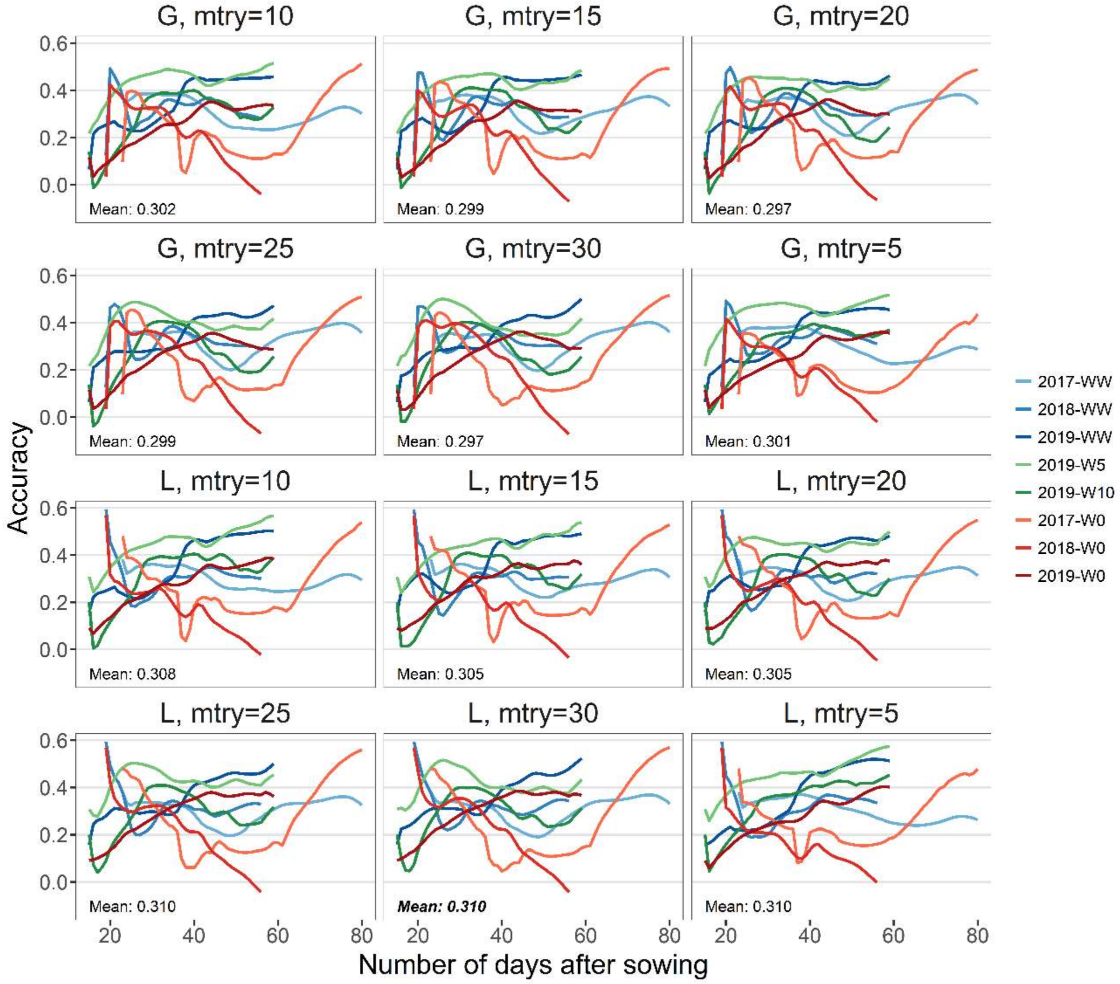

Fig S7 Prediction accuracy of the canopy height growth using the random forest model in cross-validation among genotypes and environments (CV-GE). The detail is the same as Fig. S2.

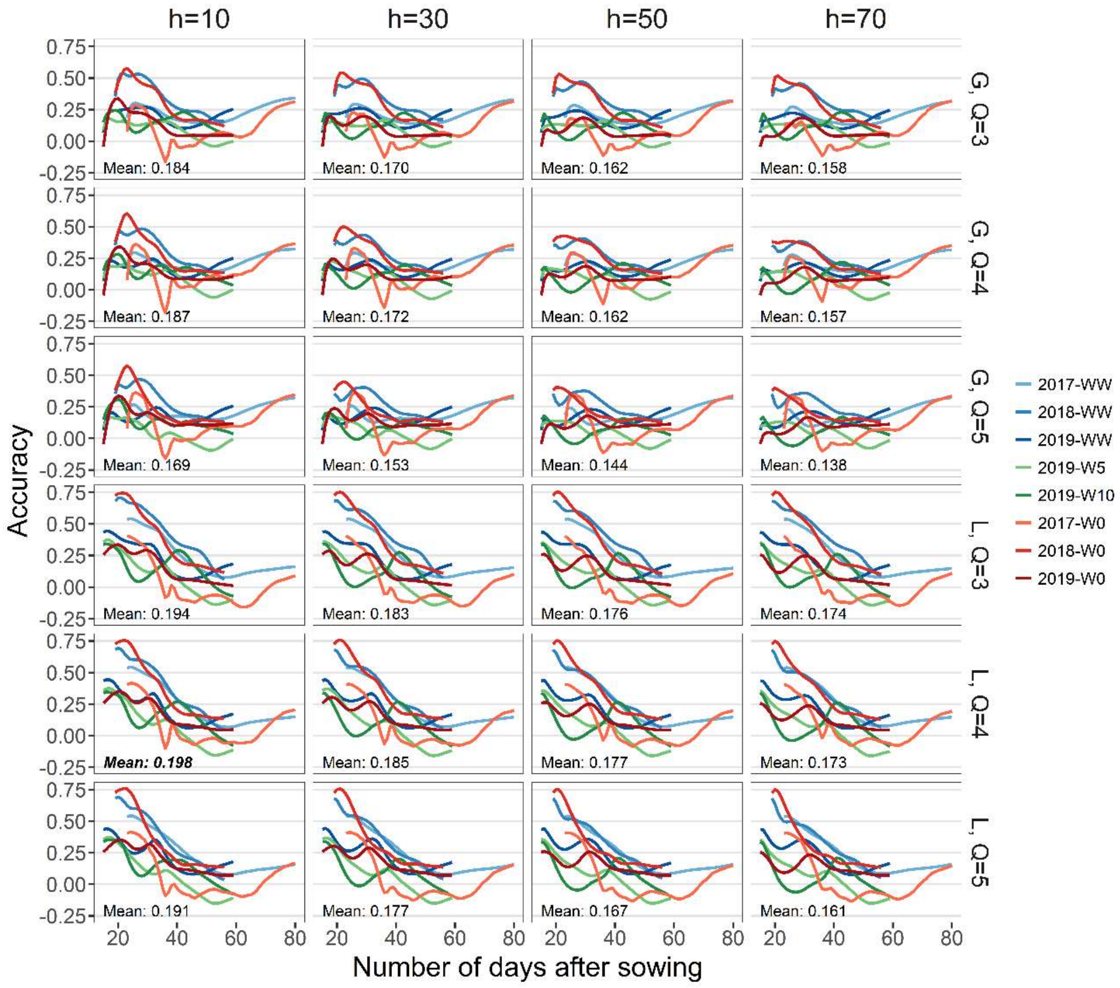

Fig S8 Prediction accuracy of the canopy area growth using the spline model in cross-validation among genotypes (CV-G). Results with different hyperparameter *h* are arranged along columns, and results with different hyperparameter *Q* and genetic relationship matrices (calculated with a linear kernel function: L or Gaussian kernel function: G) are arranged along rows. Averages of the accuracy are at the top right of each panel. The highest average of accuracy is written in bold and italic font.

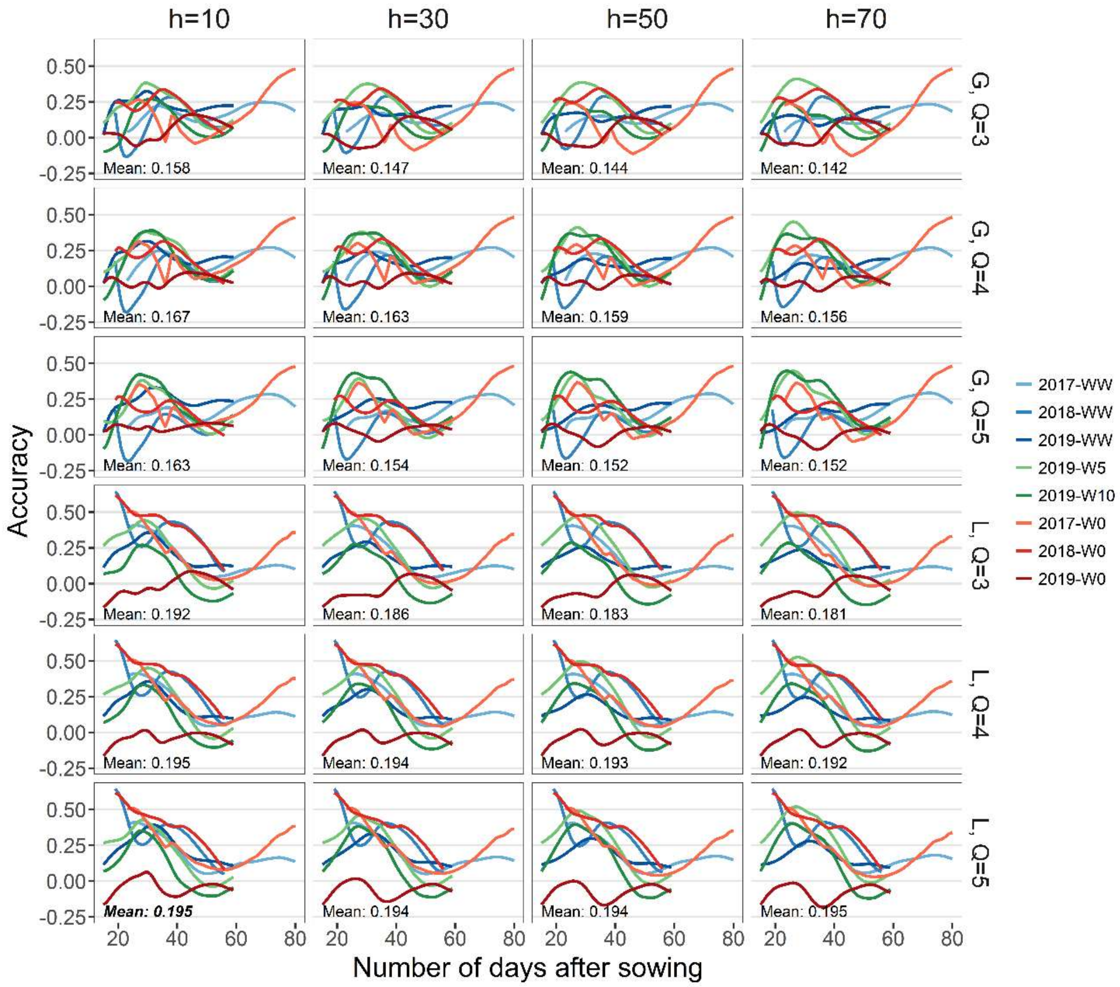

Fig S9 Prediction accuracy of the canopy height growth using the spline model in cross-validation among genotypes (CV-G). The detail is the same as Fig. S8.

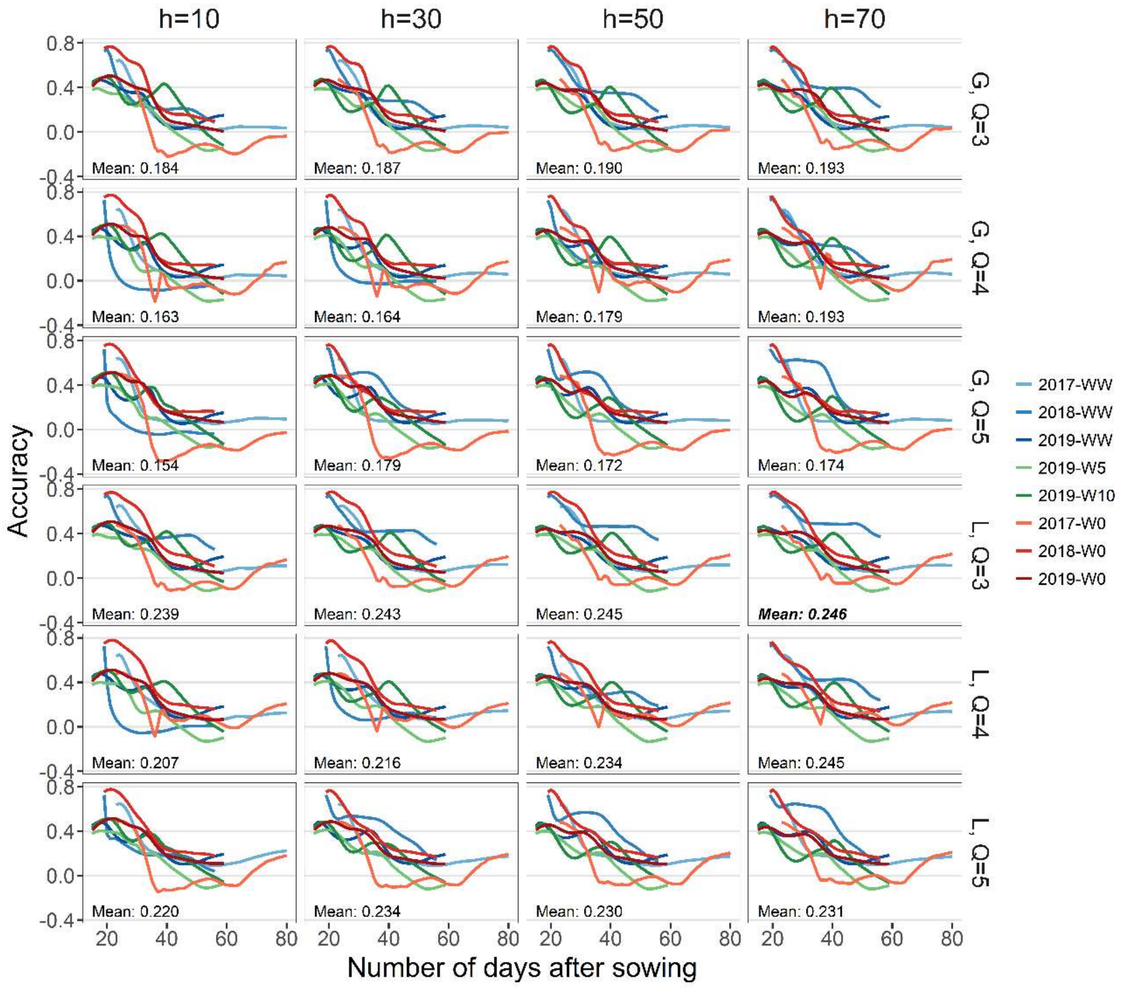

Fig S10 Prediction accuracy of the canopy area growth using the spline model in cross-validation among environments (CV-E). The detail is the same as Fig. S8.

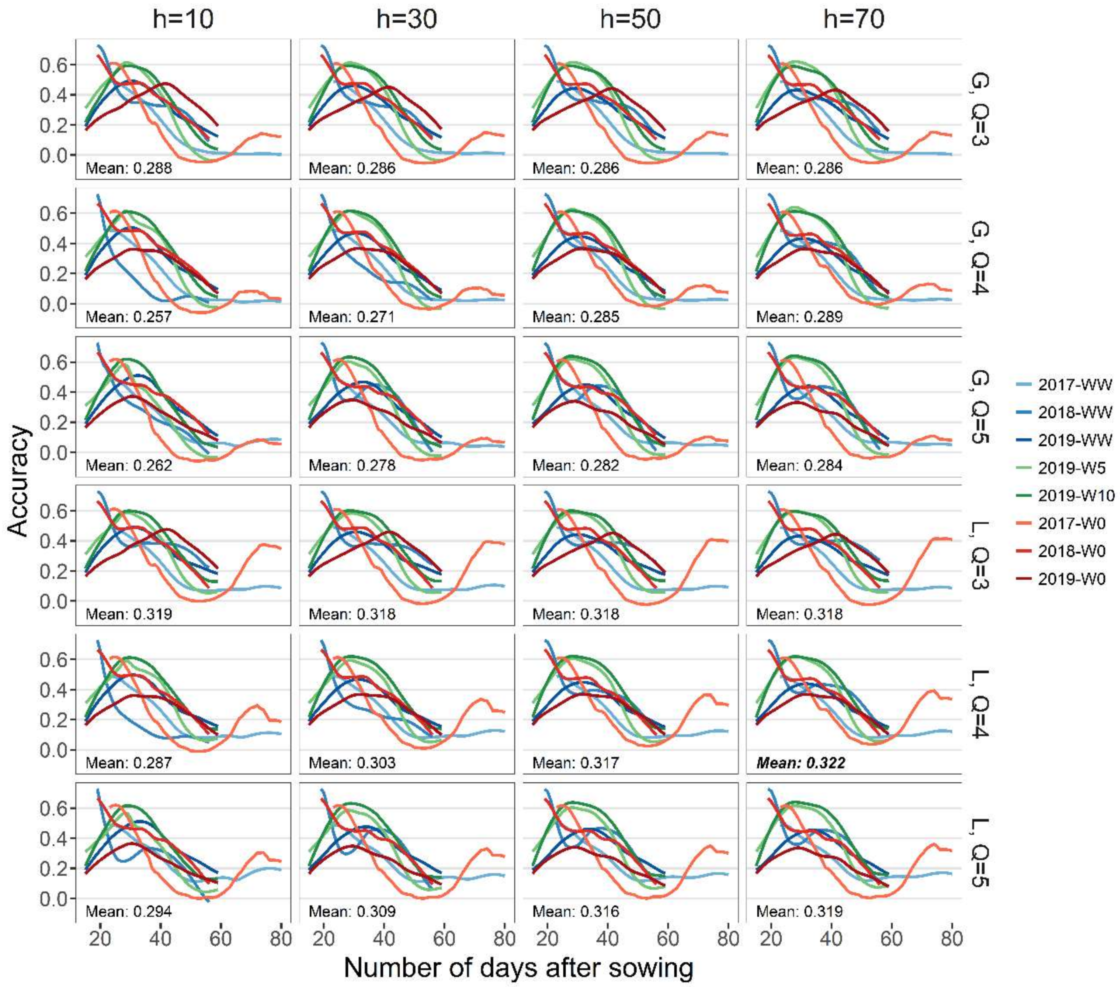

Fig S11 Prediction accuracy of the canopy height growth using the spline model in cross-validation among environments (CV-E). The detail is the same as Fig. S8.

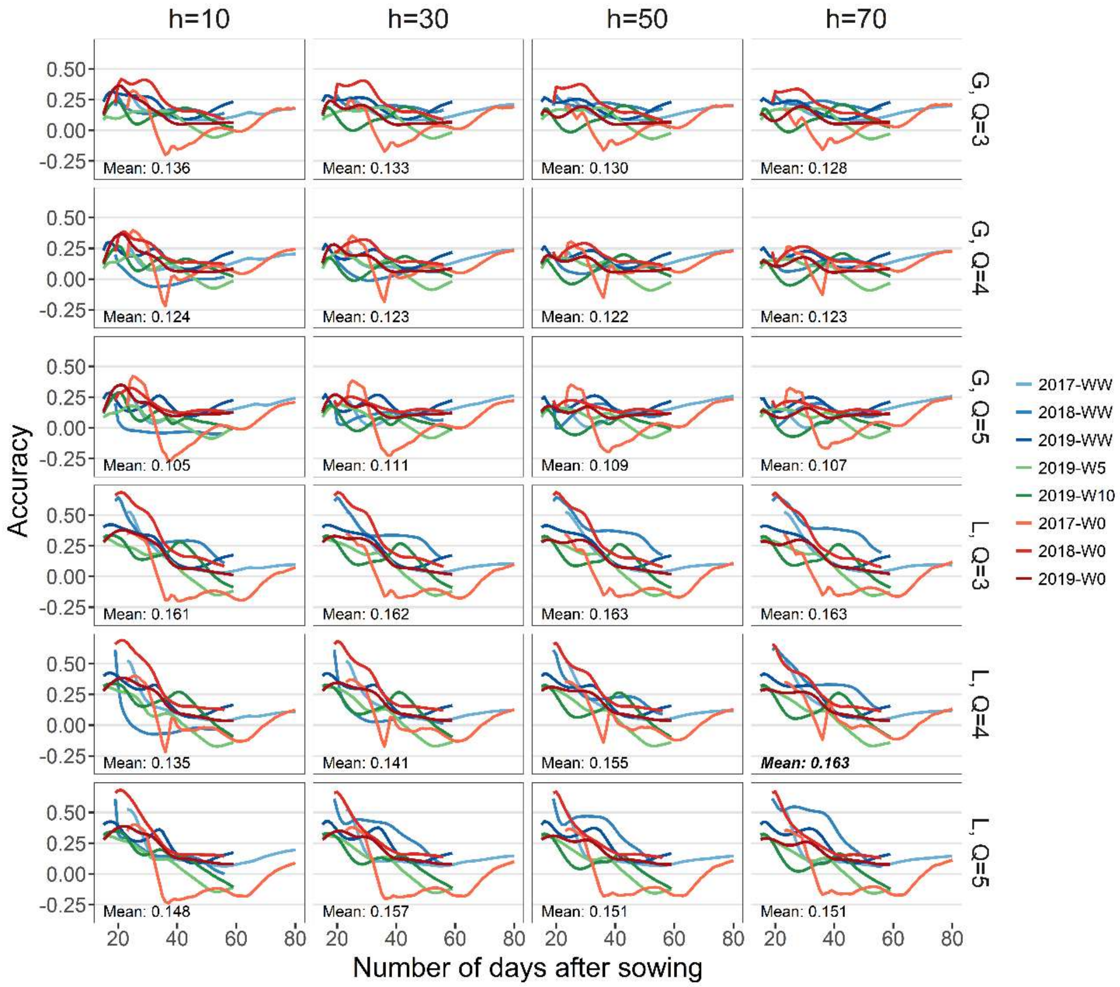

Fig S12 Prediction accuracy of the canopy area growth using the spline model in cross-validation among genotypes and environments (CV-GE). The detail is the same as Fig. S8.

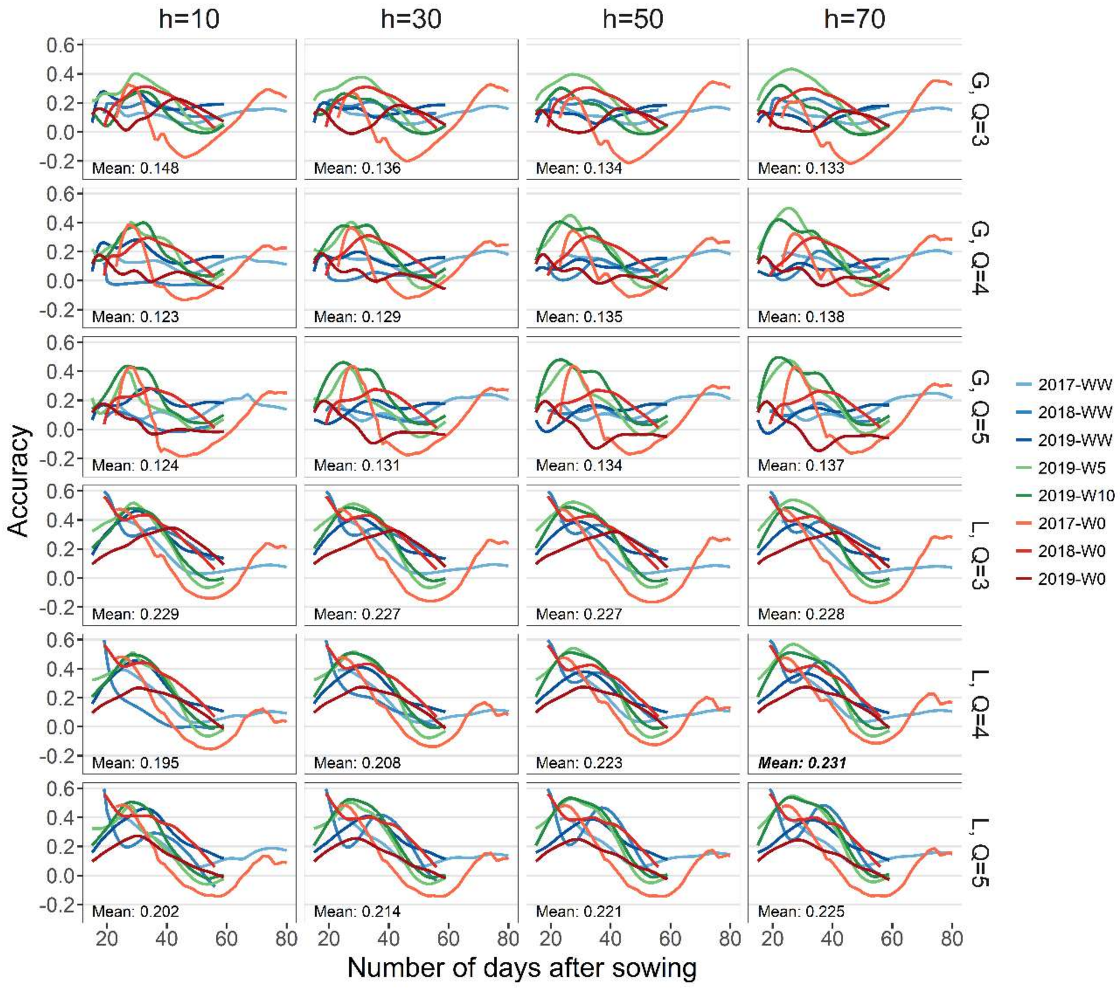

Fig S13 Prediction accuracy of the canopy height growth using the spline model in cross-validation among genotypes and environments (CV-GE). The detail is the same as Fig. S8.
